# Effects of sexual dimorphism and estrous cycle on murine *Clostridioides difficile* infection

**DOI:** 10.1128/iai.00243-26

**Published:** 2026-07-22

**Authors:** Jacqueline R. Phan, Erica Bacab, McKenzie Washington, Maria Niamba, Dung M. Do, Tiffany V. Mata, Amber Consul, Liahm Blank, Stephanie Yang, Amber J. Howerton, Efren Heredia, Robert Soriano, Michael Consul, Chandler Hassan-Casarez, Amanda M. Leisgang Osse, Maya Rae N. Mugosa, Jefferson W. Kinney, Chad L. Cross, Ernesto Abel-Santos

**Affiliations:** 1 Department of Chemistry and Biochemistry, University of Nevada Las Vegas 14722, Las Vegas, Nevada, USA; 2 Division of Infectious Diseases and Vaccinology, School of Public Health, University of California Berkeley 1438, Berkeley, California, USA; 3 Kirk Kerkorian School of Medicine, University of Nevada Las Vegas 212548 , Las Vegas, Nevada, USA; 4 Department of Physical and Life Sciences, Nevada State University 15633, Henderson, Nevada, USA; 5 Pam Quirk Brain Health Biomarker Laboratory, Department of Brain Health, Kirk Kerkorian School of Medicine 212548 , Las Vegas, Nevada, USA; 6 School of Public Health, University of Nevada Las Vegas 14722, Las Vegas, Nevada, USA; 7 Nevada Institute of Personalized Medicine, University of Nevada Las Vegas 14722, Las Vegas, Nevada, USA; University of Pennsylvania School of Veterinary Medicine, Philadelphia, Pennsylvania, USA

**Keywords:** *Clostridioides difficile *infection, CDI, sex hormones, cytokines, chemokines, immunoglobulins, sexual dimorphism

## Abstract

*Clostridioides difficile* infection (CDI) causes the majority of identifiable antibiotic-associated diarrhea. Epidemiological studies have shown that biological human females are more susceptible to CDI than males. In this study, we show that female mice developed more severe CDI than males under all conditions tested. We found time-delayed effects of the female estrus cycle on CDI. Indeed, animals in proestrus at any time during CDI progression developed severe signs 1–2 days later. In contrast, animals that were in the estrus stage were protected. Consistent with the delayed effect of the estrous cycle on CDI, we found that pre-infection levels of the sexual hormone prolactin (PRL), immunoglobulin IgG2b, cytokine IL-1β, cytokine G-CSF, and chemokine KC (CXCL1) were the primary nodes of a complex network that correlated with CDI symptomatology on the day after spore challenge. Similarly, we found that the pre-infection levels of sex hormone progesterone, sex hormone luteinizing hormone (LH), immunoglobulin IgG1, chemokine eotaxin, and chemokine IP-10 (CXCL10) were the primary network nodes that affected CDI severity, but with a 2-day delay. As expected, early post-infection levels of immunoglobulins, cytokines, and chemokines formed a hormone-independent network that concurrently correlated with CDI severity. Interestingly, early post-infection levels of FSH, together with cytokine IL-1β and chemokine KC (CXL1), were the main nodes of a network that affected CDI 2 days later, during the recovery phase of the infection. In summary, we show that murine female sexual hormones affect CDI progression, probably by affecting the immune system both before infection and during disease development.

## INTRODUCTION

*Clostridioides difficile* infection (CDI) is responsible for approximately 25% of all antibiotic-associated diarrhea (1, 2). In the United States, over 223,900 CDI cases occur annually, with a mortality rate of up to 6.5%, and costs estimated to be $6.3 billion (3–5).

When adjusted for age, immune status, and antibiotic exposure, sex is also an important risk factor in human CDI. Contrary to other GI bacterial infections (6), women are more vulnerable than men to both primary CDI and CDI relapse (7–14). Furthermore, CDI has been flagged as an emerging threat to pregnant and peripartum women (15–18). Similarly, older women might be at higher risk of developing CDI due to permanent hormonal depletion after menopause.

An obvious factor underlying sexual dimorphism is the fluctuating hormone concentrations during the female ovulation cycle (19). Sex hormones have strong effects on both human and murine metabolism (20) and can affect the immune and humoral responses (21–23), as well as susceptibility to bacterial infections (6). Changes in sex hormone levels can also affect the gut microbiota (24–26), the main protective barrier against *C. difficile* colonization (27).

Similar to humans, female mice undergo hormonal changes during the estrous cycle (Fig. S1) (28). In C57BL/6 mice, CDI-exacerbating proestrus is characterized by large increases in estradiol, LH, and FSH levels and low concentrations of prolactin (29). These spikes in hormone levels signal entry into the CDI-protective estrus. At this point, the high concentrations of circulating estradiol, progesterone, LH, and FSH characteristic of proestrus are severely diminished, followed by an increase in serum PRL. The metestrus and diestrus stages tend to have hormonal levels that fall in between those of proestrus and estrus (29). Hence, each murine estrous cycle stage is characterized by a unique pattern of hormone levels.

The characterization of the estrous cycle in mice is an approximation, as multiple studies have shown that the duration of each stage can vary significantly across various mouse strains. Similarly, the timing of hormone changes shows inter-strain variabilities (28–31). Therefore, examining hormone level shifts may provide a more quantitative approach to examining the effects of the estrous cycle on CDI.

We have previously shown that steroidal sex hormones can interact with *C. difficile* spores and modulate germination (32). Similarly, since spore germination is a necessary step for disease onset, we have found that sterane analogs can inhibit this process and prevent CDI onset in rodent models of disease (33–36). During our search for CDI prophylactics, we serendipitously observed that male mice tended to develop less severe CDI than their female counterparts.

In this study, we used a murine model to assess sexual dimorphism during CDI. We found that female mice developed more severe CDI signs than males when infected with three different *C. difficile* strains. Sexual dimorphism did not disappear under nutritional conditions known to trigger lethal murine CDI (37). Within a normal infection, CDI sign severity in female mice correlated with the estrous stages 1–2 days before maximal CDI scores. As a result of this delayed effect, animals were mostly protected from CDI if they were in estrus 1–2 days before reaching maximal signs. In contrast, animals developed deadly disease 1–2 days after proestrus. Mirroring the delayed effect of the estrous cycle on CDI, we found that pre-infection levels of PRL, immunoglobulin IgG2b, cytokine IL-1β, cytokine G-CSF, and chemokine KC were the primary nodes of a complex network that correlated with CDI symptomatology on the day after infection. Similarly, we found that the pre-infection levels of progesterone, LH, immunoglobulin IgG1, chemokine eotaxin, and chemokine IP-10 were the primary network nodes that affected CDI severity, but with a 2-day delay. As expected, the levels of immunoglobulins, cytokines, and chemokines during early infection formed a hormone-independent network that concurrently correlated with CDI severity. Interestingly, early infection levels of FSH, together with cytokine IL-1β and chemokine KC, were the main nodes of a network that affected CDI severity 2 days later, during the recovery phase of the infection. In summary, we show that murine female sexual hormones affect CDI progression, likely as a function of immune system modulation, both before infection and during disease progression.

## MATERIALS AND METHODS

### Materials

*C. difficile* strains R20291 and 9001966 were donated by Professor Nigel Minton at the University of Nottingham, United Kingdom (38). *C. difficile* strain 630 was obtained from the American Type Culture Collection (ATCC, Manassas, VA) (Table S1). The synthetic bile salt analogs, CamSA and CaPA, were prepared by Professor Steven M. Firestine at Wayne State University in Detroit, Michigan. Standard Laboratory Rodent Diet was obtained from LabDiet (St. Louis, MO, USA). Custom purified diet (Atkins-type diet) containing approximately 50% protein and 25% fat was purchased from TestDiet (TestDiet Catalog # 18181230286). All *C. difficile* strains were grown and maintained in a Coy Laboratories (Grass Lake, MI) vinyl anaerobic chamber (5% H_2_, 5% CO_2_, 95% N_2_).

### Animals

Weaned C57BL/6 mice were purchased from Charles River Laboratories (Wilmington, MA, USA). Mice were housed in groups of five per cage at the University of Nevada, Las Vegas, animal care facility. Upon arrival at the facility, animals were allowed to acclimate for at least one week before the start of experimentation. All post-challenge manipulations were performed within a biosafety level 2 laminar flow hood.

### *C. difficile* spore harvest and purification

*C. difficile* cells from frozen stocks were streak plated in an anaerobic chamber onto BHI agar supplemented with 2% yeast extract, 0.1% L-cysteine-HCl, and 0.05% sodium taurocholate (BHIS) to yield colonies (15). After 48 h, a single colony was inoculated into BHI broth supplemented with 0.5% yeast extract and incubated for 48 h. The inoculated broth was then spread-plated onto multiple BHI agar plates prepared as described above. Inoculated plates were incubated for 7 days at 37°C.

Plates were then flooded with ice-cold deionized (DI) water. Cells and spores were harvested by scraping bacterial colonies from the plates. The cell and spore mixtures were pelleted by centrifugation at 8,000 × *g* for 5 min, resuspended in DI water, and pelleted again. This washing step was repeated twice more. The cell/spore mixture was then centrifuged through a 20% to 50% HistoDenz gradient at 18,200 × *g* for 30 min without braking (22). Under these conditions, spores pelleted at the bottom of the centrifuge tube, while cell debris remains above the 20% HistoDenz layer. Pelleted spores were transferred to a clean centrifuge tube and washed five times before storing in DI water at 4°C. To determine spore purity, selected samples were stained using the Schaeffer-Fulton endospore staining method (23) or were visualized via phase-contrast microscopy. Spore preparations used were greater than 95% pure. Spores were washed three times with DI water, heat-shocked at 68°C for 30 min, then washed three additional times before being resuspended in DI water.

### Estrous cycle tracking in mice

Tracking of the estrous cycle was adapted from McLean et al. (29). Mice’s estrous stages were tracked for at least 1 week before any manipulation to ensure that animals were cycling normally. Animals were tracked daily for their estrous cycle stage via vaginal lavage at the same time each morning throughout this study. First, excess urine is dabbed off with low-lint Kimwipes. A micropipette was used to lavage 20 μL of 1× phosphate-buffered saline (PBS) into the vaginal orifice of the animal. PBS was swooshed 3–4 times around the vaginal orifice until the suspension appeared mildly cloudy. The 20-μL sample was then transferred onto a clean glass microscope slide and air-dried for at least 1 h. Animals were also monitored to ensure there were no signs of vaginal infection.

Air-dried vaginal sample slides were dipped into a 2% crystal violet solution inside a Coplin jar for 1 min. Slides were then moved to a Coplin jar containing fresh DI water to wash off excess stain. Slides were dabbed gently with clean wipes, allowed to air dry, and examined using a compound light microscope at 100× and 400× magnification (Fig. S2) (39).

### Murine CDI model

The murine CDI model used in this study was adapted from Chen et al. (40). Mice were given three consecutive days of an antibiotic cocktail containing kanamycin (0.4 mg/mL), gentamycin (0.035 mg/mL), colistin (850 U/ml), metronidazole (0.215 mg/mL), and vancomycin (0.045 mg/mL) *ad libitum* (14). Mice were then given DI water for the remainder of the experiment. On the day before *C. difficile* challenge (day −1), mice were given an intraperitoneal (IP) injection of 10 mg/kg clindamycin. On the day of infection (day 0), experimental groups were challenged with 10^8^ *C. difficile* spores via oral gavage.

Mice were observed for signs of CDI twice daily, and disease severity was scored according to a CDI sign rubric adapted from previously published work (34). According to the rubric, pink anogenital area, mild wet tail, and weight loss of 8%–15% were each given a score of 1. Red anogenital area, lethargy/distress, increased diarrhea/soiled bedding, hunched posture, and weight loss greater than 15% were each given a score of 2. Scores were tallied to give a final severity score. Animals with a cumulative score of less than three were indistinguishable from noninfected controls and were considered non-diseased. Animals scoring 3–4 were considered to have mild CDI. Animals scoring 5–6 were considered to have moderate CDI. Animals scoring greater than 6 were considered to have severe CDI and were immediately culled.

### Tracking the estrous cycle in the murine CDI model

To better visualize the diverse range of murine CDI signs, the hypervirulent strain R20291 was employed for all estrous-related studies. Estrous stages for each mouse were tracked every morning at the same time via vaginal lavage and cytology. Mice that were not cycling or cycling irregularly were excluded from the study. In the first round of the study (cohort 1), animals were sacrificed at random on day 0 (prior to *C. difficile* challenge), day 1, day 2, and day 3 (post-*C. difficile* challenge) after daily estrous stage sampling. Animals that were severely ill were immediately culled regardless of day or estrous stage. Surviving animals on each day were analyzed for estrous stage and CDI signs.

After initial analysis of data from the first experiments showed that estrous stages have a delayed effect on CDI severity, the protocol was further modified in subsequent experiments to specifically track specific estrous stages and collect non-terminal blood samples on the days before expected maximal CDI signs. In the second round (cohort 2), mice were pooled based on their predicted estrous stage on the day of spore challenge. This prediction was estimated from the estrous stage they were at the start of antibiotic treatment, five days before infection. For each estrous stage grouping, animals were randomly selected for blood sampling via facial vein bleed on day 0 (day of challenge) or day 1 (24 h after challenge). Animals were monitored for CDI signs, as above, and sacrificed either one day or two days after blood samples were collected.

### Blood sample processing for sex hormones, cytokines/chemokines, and immunoglobulins

Approximately 100 μL of blood was collected from each mouse via facial vein bleeds. The blood rested at room temperature for 30 min before spinning down to collect serum. Serum was frozen at −20°C and then stored at −80°C until processing. Each serum sample was divided into four aliquots.

The first aliquot was analyzed to determine the concentrations of follicle-stimulating hormone (FSH), luteinizing hormone (LH), and prolactin (PRL) using the Milliplex Mouse Pituitary Magnetic Bead Panel from Millipore according to kit instructions on a Bio-Plex 200 system. The second aliquot was processed using Millipore’s Milliplex Mouse Cytokine/Chemokine Magnetic Bead Panel according to kit instructions to test for cytokines IL-13, IL-10, IL-2, IP-10, IL-5, RANTES, KC, IL-15, IL-1α, IL-1β, IL-17, IL-6, TNF-α, G-CSF, eotaxin, IFN-γ, and GM-CSF. The third aliquot was processed using Milliplex Mouse Immunoglobulin Isotyping Magnetic Bead Panel from Millipore according to kit instructions to test for IgM, IgG, IgA, IgE, and IgD.

The fourth aliquot was sent to The Metabolomics Innovation Center (TMIC, Alberta, Canada) for analysis of progesterone concentrations by HPLC-MS. Briefly, 10 μL of plasma was extracted with 40 μL of acetonitrile containing 3.1 nM progesterone-d9 internal standard (Cayman Chemical, Ann Arbor, MI). The extraction mixture was sonicated in a water bath for 10 min, centrifuged at 21,000 × *g* for 1 min, and 40 μL of the supernatant was transferred to autosampler vials with glass inserts. LC-MRM-MS analysis was performed using a Nexera XR UPLC system (Shimadzu, Japan) coupled to a Sciex QTrap 6500 + mass spectrometer equipped with an IonDrive Turbo V electrospray ionization source. Chromatography was performed using a Zorbax Eclipse Plus C18 column (2.1 × 150 mm, 1.8 μm, Agilent, USA) at a flow rate of 0.2 mL/min of mobile phases (A) 0.1% formic acid in water and (B) 0.1% formic acid in acetonitrile. The LC gradient was 50%–100% mobile phase B (0.0–5.0 min), 100%–50% mobile phase B (5.0–5.1 min), and 50% mobile phase B (5.1–8.0 min). The sample injection volume was 10 μL, the column oven was off, and the autosampler temperature was 15°C. MRM-MS was performed using electrospray ionization in the positive mode. Then, 315.2/97.1 and 324.3/100.1 Q1/Q3 transitions were monitored for progesterone and progesterone-d9, respectively (1). Dwell time was 50 ms, collision energy (CE) 32 V, declustering potential (DP) 50 V, entrance potential (EP) 10 V, collision cell exit potential (CXP) 12 V, source temperature 450°C; ion source gas 1 40 psi; ion source gas 2 40 psi. The collision gas was set to high, and the curtain gas was set to 40 psi. Data acquisition and analysis were performed using Analyst 1.6.3 software (Sciex, Framingham, MA). Progesterone concentrations were determined from chromatographic peak area ratios of the analyte and the internal standard.

### Statistical analyses

For the murine CDI model, severity of signs was visualized via violin plots with a minimum of three independent values (*n* ≥ 3) using GraphPad Prism v.10. One-way ANOVA or one-tailed *t*-test was performed, as appropriate, using R (v. 4.4.3) (41) to assess differences between groups at every time point, with significance defined as *P* < 0.05. Significant main-effect ANOVAs were analyzed using Scheffe’s correction for *post hoc* comparisons between all groups. Estrous stages with only one mouse were not included in the statistical analysis. Time-to-event data were analyzed using Kaplan-Meier survival analysis models, with log-rank tests for curve comparisons. Alphabet letters denote statistically similar groups. For the 24- and 48-hour data sets combined across experiments, CDI severity (CDI max) was analyzed using a General Linear Model (GLM) in IBM SPSS Statistics (v. 26), with estrous stage (four-level categorical variable: proestrus, estrus, metestrus, diestrus) as a fixed factor and batch (Experimental Cohort 1 vs Experimental Cohort 2) included as an additional factor to account for differences between experiments. Analyses were performed separately for each time point (24 and 48 h). To account for uncertainty in estrous staging, samples with ambiguous classifications (e.g., transitional stages such as proestrus/estrus or early/late designations) were identified and reassigned to one of the four primary stages (proestrus, estrus, metestrus, diestrus) based on the later stage in the cycle (e.g., proestrus/estrus and early estrus were categorized as estrus, while early proestrus and late diestrus were categorized according to their respective stages). This approach was used because transitional cytological classifications represent intermediate stages within a continuous and directional estrous cycle, and assignment to the subsequent stage provides a consistent framework for categorization. Analyses were then performed both including all animals with these re-binned classifications and excluding ambiguous cases as a sensitivity check. When significant main effects were detected, *post hoc* pairwise comparisons were performed using Bonferroni correction.

For assessment of associations among variables, we utilized the robust Kendall’s Tau correlation coefficient for bivariate comparisons among hormone levels, cytokine levels, immunoglobulin levels, and CDI severity scores, with significance defined as *P* < 0.10. Correlations were also calculated for combined samples and individually by bleed day (day 0 or day 1). The complete set of correlation analyses is shown in Tables S2 to S11. Positive correlations are highlighted in green. Negative correlations are highlighted in red. Dark colors represent strong correlations. Medium colors represent moderate correlations. Light colors represent weak correlations. Lack of correlations is shown as non-highlighted.

## RESULTS

### Female mice exhibit more severe CDI than male mice

**Fig 1:**
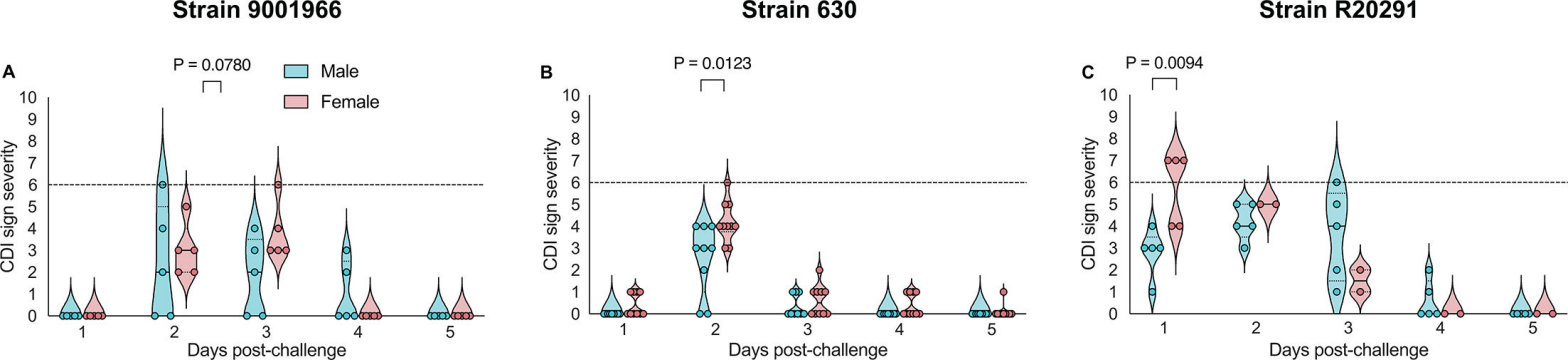
Effect of sex on CDI severity. Violin plots of CDI sign severity in female mice (pink) and male mice (blue) challenged with *C. difficile* spores from (**A**) strain 9001966 (female = 5; male *n* = 5), (**B**) strain 630 (female *n* = 10; male *n* = 9), and (**C**) strain R20291 (female *n* = 5; male *n* = 5). Signs of severity were determined daily based on the CDI scoring rubric reported previously (34). Animals with a score of <3 were indistinguishable from noninfected controls and were considered non-diseased. Animals with a score of 3 to 4 were considered to have mild CDI. Animals with a score of 5 to 6 were considered to have moderate CDI. Animals with a score of >6 were considered to have severe CDI and were immediately culled. The long-dashed line represents the clinical endpoint. Each individual mouse is denoted by a circle. Horizontal solid lines represent median values, dotted lines represent interquartile ranges, and tapered ends represent maximum and minimum values. Daily statistical differences between males and females were determined by one-tailed unpaired *t*-test.

We infected male and female C57BL/6 mice with three different *C. difficile* strains (34). These strains represent different ribotypes and were selected because they show distinct timelines for CDI onset. One group of male and female mice was infected with spores from *C. difficile* strain 9001966 (ribotype 106), a clinical strain from the United Kingdom and shows delayed onset of murine CDI (38) (Fig. 1A). For both sexes, mice started to develop signs of CDI on day 2 post-challenge (day 2). Male mice showed varied CDI sign severities during days 2–4 (CV_day 2_ = 108.7%; CV_day 3_ = 99.4%; CV_day 4_ = 141.4%), but all animals recovered by day 5. Female mice, on the other hand, showed more homogeneous mild to moderate CDI symptomatology in days 2–3 (CV_day 2_ = 40.8%; CV_day 3_ = 34.3%) and all animals recovered by day 4. Even though the CDI timeline was shorter in female mice, these animals developed statistically more severe signs than their male counterparts at the peak of infection (Fig. 1A).

A second group of male and female mice was infected with spores from *C. difficile* strain 630 (ribotype 012), a type strain (42, 43) that we have extensively tested for its capacity to induce CDI symptoms in mice. Male mice infected with *C. difficile* strain 630 spores showed less individual variability in CDI symptomatology and shorter infection progression compared with animals infected with *C. difficile* strain 9001966. Both male and female mice infected with *C. difficile* strain 630 did not show any signs on day 1 after challenge (Fig. 1B). By day 2, female mice showed a notable increase in CDI sign severity (CDI_avg_ = 4.2; CV_day 2_ = 21.9%), followed by a return to mild CDI signs by day 3 (CDI_avg_ = 0.6; CV_day 3_ = 116.5%), and complete remission by day 5 post-challenge. Similar to female mice, CDI signs for male mice started to resolve by day 3 (CV_day 2_ = 62.2%; CV_day 3_ = 150%) and disappeared by day 5 post-challenge. In both male and female mice infected with *C. difficile* strain 630, CDI sign severity peaked on day 2 post-challenge. Notably, male mice showed mostly mild-to-moderate sign severity, while female mice showed statistically more severe symptomatology (male CDI_max_ = 2.6 vs female CDI_max_ = 4.2) (Fig. 1B).

A third group of male and female mice was infected with spores from the hypervirulent *C. difficile* strain R20291 (ribotype 027), a hypervirulent strain that causes earlier infection onset and more severe murine CDI signs than other strains (34). Male mice challenged with *C. difficile* strain R20291 started to develop moderate CDI symptoms by day one post-spore challenge (CDI_avg_ = 2.6; CV_day 1_ = 39.12%) (Fig. 1C). Male mice remained moderately symptomatic until day 4 (CDI_avg_ = 0.6; CV_day 4_ = 149.1%), when CDI signs started to resolve. Similar to animals infected with *C. difficile* strain 9001966 and *C. difficile* strain 630, all male mice infected with *C. difficile* strain R20291 recovered and showed no CDI signs by day 4.

**Fig 2:**
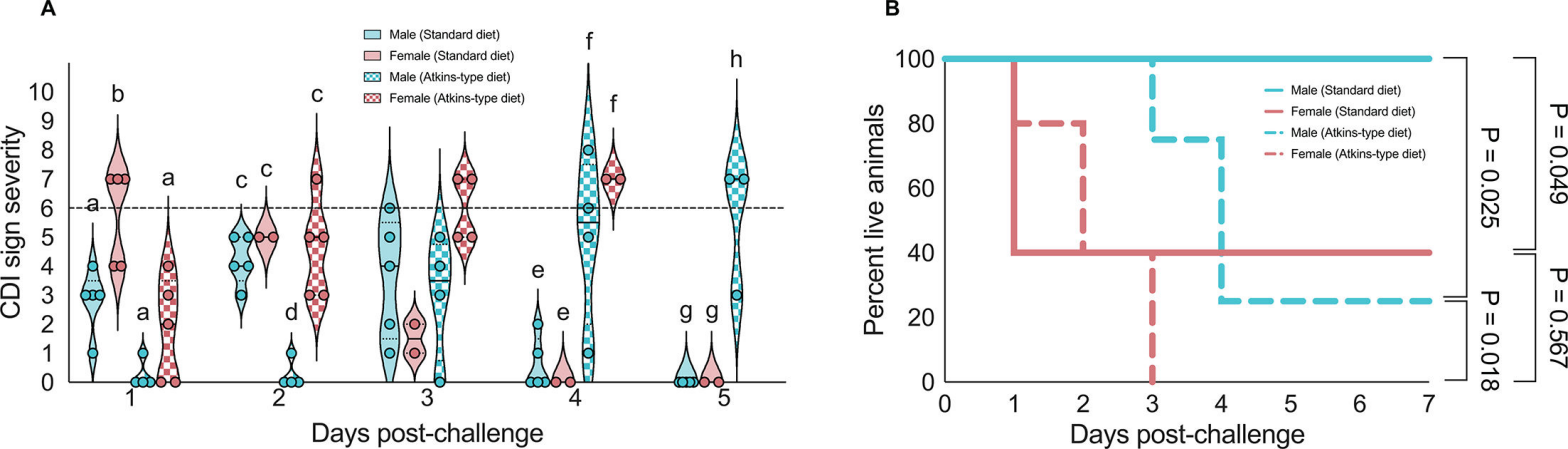
Effect of Atkins-type diet on mice CDI survival. (**A**) Violin plots of CDI sign severity for male mice fed a standard diet (*n* = 5; solid blue), female mice fed a standard diet (*n* = 5; solid pink), male mice fed an Atkins-type diet (*n* = 4; blue checker), and female mice fed an Atkins-type diet (*n* = 5; pink checker) challenged with *C. difficile* spores from strain R20291 (34). Data for mice fed the standard diet are the same as shown in Fig. 1C. The long-dashed line represents the clinical end point. Each individual mouse is denoted by a circle. Horizontal solid lines represent median values, dotted lines represent interquartile ranges, and tapered ends represent maximum and minimum values. Single-factor ANOVA was performed at every time point to assess differences among the sign severity means of the different groups. ANOVA results with *P* values of <0.05 were analyzed *post hoc* using the Scheffe test for pairwise comparison between daily groups. Columns that are labeled with different letters are statistically different. Columns that are labeled with the same letter are statistically similar. (**B**) Kaplan-Meier survival plots for male mice fed a standard diet (solid blue line), female mice fed a standard diet (solid pink line), male mice fed an Atkins-type diet (dashed blue line), and female mice fed an Atkins-type diet (dashed pink line) challenged with *C. difficile* spores from strain R20291. Statistical survival comparisons of different groups were performed via log-rank test.

In contrast, female mice challenged with *C. difficile* strain R20291 spores developed statistically more severe CDI signs than male mice the day after spore challenge (male CDI_max_ = 4.2 vs female CDI_max_ = 5.8) (Fig. 1C). Indeed, while all males infected with *C. difficile* strain R20291 recovered from CDI, more than half of the female cohort succumbed to the infection (Fig. 2B, *P* = 0.049). Surviving female mice started to slowly recover from CDI and showed no symptoms by day 4. Because animals challenged with *C. difficile* strain R20291 developed the most severe CDI signs, we used this hypervirulent strain in subsequent experiments.

### A high-protein/high-fat (Atkins-type) diet aggravates CDI in mice of both sexes

We recently showed that an Atkins-type diet can exacerbate CDI severity in mice (37). To determine if sex affects CDI exacerbation by diet, male and female mice challenged with *C. difficile* strain R20291 while on this high-protein/high-fat (Atkins-type) diet showed delayed onset of CDI signs compared with animals fed a standard diet (Fig. 2A). Although female mice fed an Atkins-type diet did not show statistically more severe CDI signs (Fig. 2A) or survival (Fig. 2B) than female mice fed a standard diet, by day 4 post-challenge, all female mice in the Atkins-type diet had developed severe CDI and had to be culled.

In contrast, once the infection started, male mice fed an Atkins-type diet developed statistically more severe CDI signs compared with challenged mice fed a standard diet (Fig. 2A). On a standard diet, 100% of males survived compared with 40% of females (*P* = 0.049). Indeed, while all male mice on a standard diet survived CDI, 75% of male mice fed an Atkins-type diet developed signs severe enough to require euthanasia (Fig. 2B). In fact, survival for male mice on an Atkins-type diet was statistically worse than male mice fed a standard diet (*P* = 0.025), comparable to female mice fed a standard diet, but still better than females on the Atkins-type diet (*P* = 0.018). In both cases, mice fed the Atkins-type diet appeared to exhibit lingering CDI, whereas mice fed a standard diet tended to recover more following day 3 post-infection.

### Estrous stages have a delayed effect on CDI severity prior to infection

**Fig 3:**
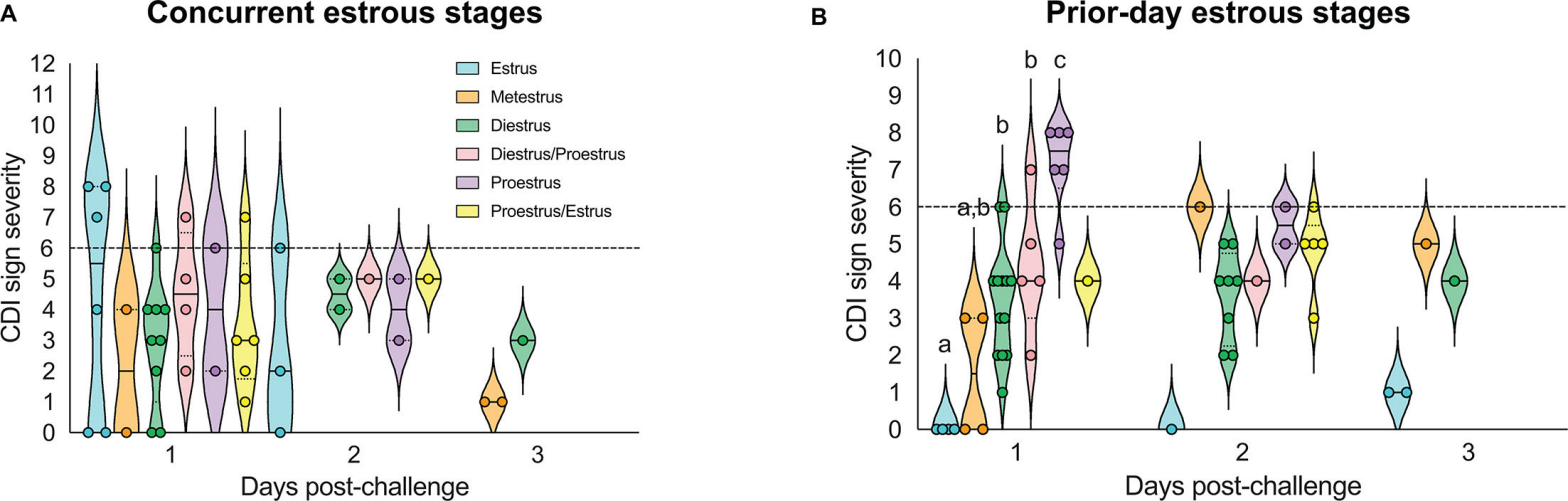
Time-dependent effect of estrous cycle on daily CDI severity. Violin plots of daily CDI sign severity plotted against (**A**) their concurrent estrous stages and (**B**) their prior-day estrous stages. Animals in estrus (blue), metestrus (orange), diestrus (green), late diestrus/early proestrus (pink), proestrus (purple), and late proestrus/early estrus (yellow) were challenged with *C. difficile* spores from strain R20291 on day 0. CDI sign severity and estrous cycle stages were determined daily. Signs of severity were determined based on the CDI scoring rubric reported previously (34). The long-dashed line represents the clinical end point. The number of animals in each cycle varies as each individual animal progresses through the estrous cycle. Each individual mouse is denoted by a circle. Horizontal solid lines represent median values, dotted lines represent interquartile ranges, and tapered ends represent maximum and minimum values. Single-factor ANOVA was performed at every time point to assess differences among the sign severity means of the different estrous stages. ANOVA results with *P* values of <0.05 were analyzed *post hoc* using the Scheffe test for pairwise comparison between daily groups. Columns that are labeled with different letters are statistically different. Columns that are labeled with the same letter are statistically similar. Estrous stages with only one mouse were not included in the statistical analysis.

Using vaginal smear cytology (Fig. S1), we were able to monitor the estrous cycle of female mice. Animals were binned (Fig. 3) into estrus stage (blue), metestrus stage (orange), diestrus stage (green), and proestrus stage (purple). Some animals could not be unambiguously assigned to a specific estrous stage. These animals showed borderline cytology readings and were thus binned into two new categories: late diestrus/early proestrus (pink) and late proestrus/early estrus (yellow).

To determine the effect of the estrous cycle on murine CDI severity, a first set of female mice in distinct stages of the estrous cycle were challenged with *C. difficile* strain R20291 spores. We continued to simultaneously monitor the estrous cycle and CDI sign progressions. As the estrous cycle does not stop during the course of infection, animals could cycle across different estrous stages while CDI signs are being monitored. As a first approach, we tried to correlate daily CDI signs for each animal with their concurrent estrous stage, but we found no statistical significance between groups (Fig. 3A).

Intriguingly, when daily CDI severity was plotted against the estrous stages of the prior day, we found distinct daily patterns (Fig. 3B). The strongest statistical correlation between CDI signs and prior day estrous stages was found on day one post-challenge. Animals that were in estrus when challenged with *C. difficile* spores (day 0) showed no CDI signs the following day (day 1). Compared with animals that were in estrus on day 0, animals that were in metestrus, diestrus, and late diestrus/early proestrus during spore challenge developed statistically higher CDI severity the following day. Finally, animals that were at proestrus at day 0 developed the most severe CDI symptoms the day after challenge, with more than half reaching the clinical endpoint.

It should be noted that in both approaches, the number of mice in each estrous stage varied greatly. Although staging in days before infection provides estimates for stages on subsequent days, individual mice did not strictly follow the same timeline through estrous, even when co-housed. Additionally, fewer animals overall were staged beyond day 1 post-challenge due to lower numbers of mice left after culling on previous days.

The effect of prior-day estrous stages on CDI sign severity started to dissipate by day 2 post-challenge. Unfortunately, there was an insufficient number of animals in the proestrus and the late diestrus/early proestrus groups on day 1 for a complete statistical analysis of CDI severity on day 2 post-challenge. Furthermore, because of the high CDI-mediated mortality on days 1 and 2, there were insufficient numbers of animals left for statistical analysis on CDI score on day 3 post-challenge versus estrous stages.

**Fig 4:**
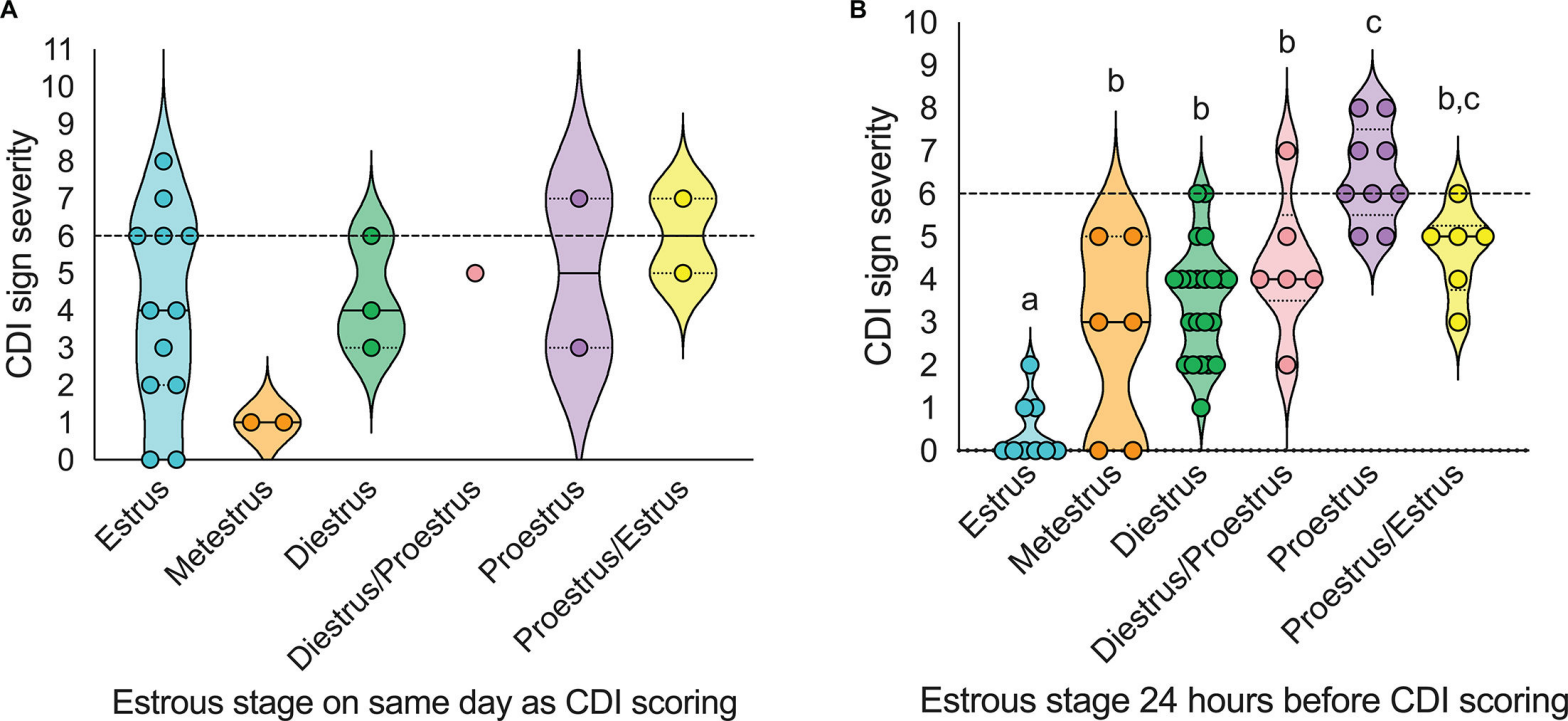
Time-independent effect of estrous cycle on CDI severity. Cumulative violin plots of CDI sign severity caused by *C. difficile* strain R20291 plotted against (**A**) their concurrent estrous stages, and (**B**) their prior-day estrous stages. Animals that were at estrus (blue), metestrus (orange), diestrus (green), late diestrus/early proestrus (pink), proestrus (purple), and late proestrus/early estrus (yellow) at any day were pooled together. CDI sign severity and estrous cycle stages were determined daily. Signs of severity were determined based on the CDI scoring rubric reported previously (34). The long-dashed line represents the clinical endpoint. The number of animals in each cycle varies as each individual animal progresses through the estrous cycle. Each individual mouse is denoted by a circle. Horizontal solid lines represent median values, dotted lines represent interquartile ranges, and tapered ends represent maximum and minimum values. Single-factor ANOVA was performed to assess differences among the sign severity means of the different estrous stages. ANOVA results with *P* values of <0.05 were analyzed *post hoc* using the Scheffe test for pairwise comparison between all groups. Columns that are labeled with different letters are statistically different. Columns that are labeled with the same letter are statistically similar.

To determine if the prior-day estrous effect is independent of the infection time course, we combined the data for each estrous stage regardless of the day they were determined. This allowed us to increase the number of animals in each estrous stage for statistical analysis. As expected, plotting CDI severity against their concurrent estrous stages showed no statistically significant differences (Fig. 4A).

However, when CDI severity for each animal was plotted against their prior-day estrous stage, a similar delayed pattern was observed as in Fig. 3B. Indeed, when animals were in estrus at any day during infection, they showed few CDI signs the next day (Fig. 4B). Animals that were in metestrus, diestrus, and diestrus/proestrus at any time showed more varied but similar intermediate symptomatology one day later. In contrast, animals in proestrus at any time during infection became extremely sick the next day and had to be culled. Any animals in proestrus/estrus showed next-day CDI severity intermediate between proestrus and estrus.

### Estrous stages are correlated with the day of maximal CDI sign severity

During the previous part of the study, only vaginal lavage samples were collected and examined to determine the estrous stage of animals. To further investigate the delayed effect of the estrous stages on CDI severity, a second cohort of mice was tracked and timed for blood collection and sacrificed based on their predicted estrous stage on the day of spore challenge with hypervirulent strain R20291. Instead of collecting terminal blood samples, facial vein blood samples were taken 1–2 days before sacrifice. Contrary to expectations, mice did not develop maximal CDI signs 1-day post-challenge as the previous cohort of mice (Fig. 1A). Instead, all mice developed the most severe CDI signs 2 days after infection. Consequently, no correlation was observed between estrous stage (via vaginal lavage and cytology) and day 1 post-challenge.

**Fig 5:**
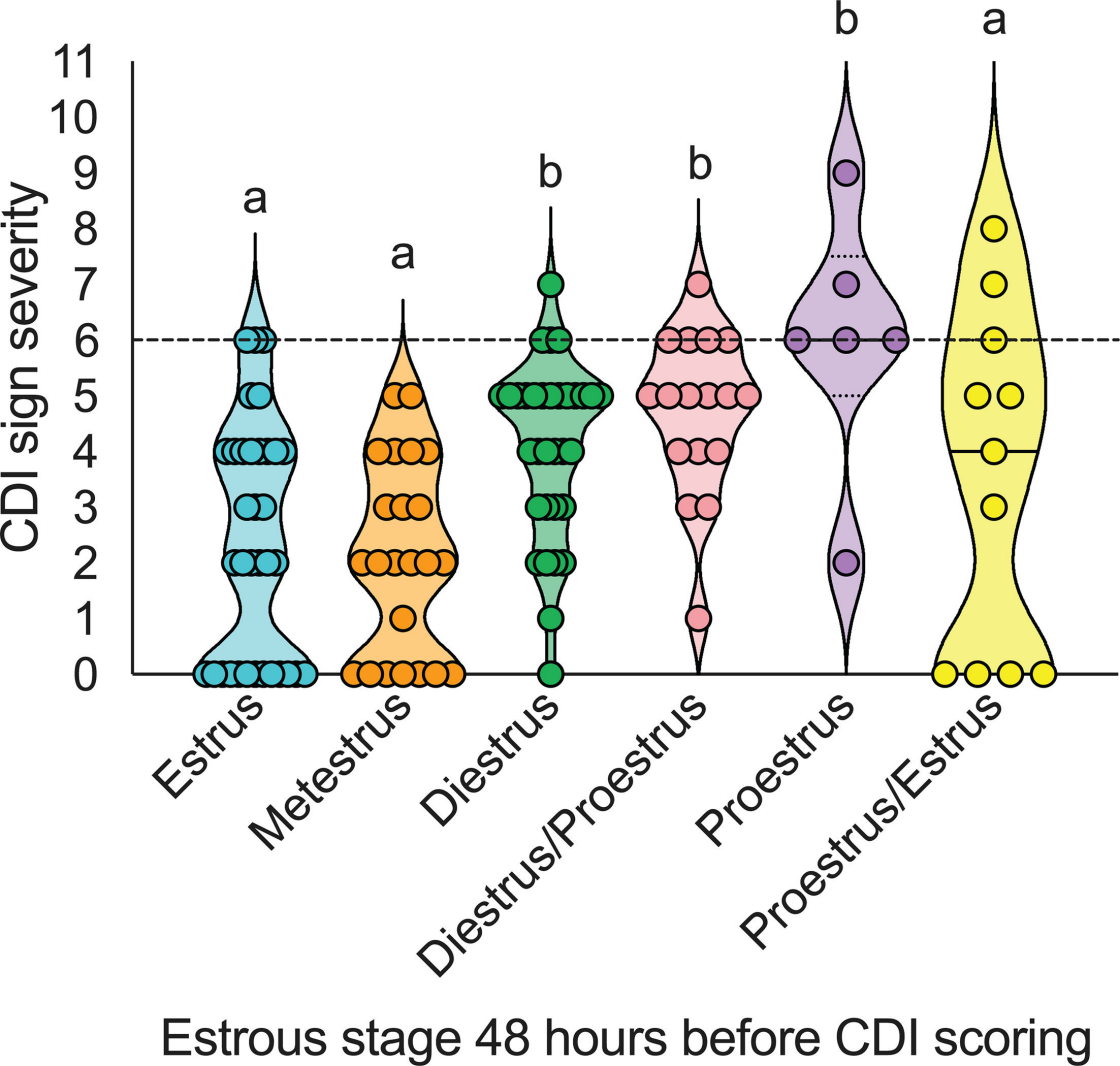
CDI severity score and estrous stage 48 h prior. Cumulative violin plots of CDI sign severity caused by *C. difficile* strain R20291 plotted against their 2-day prior estrous stages. CDI sign severity and estrous cycle stages were determined daily. Signs of severity were determined based on the CDI scoring rubric reported previously (34). The long-dashed line represents the clinical endpoint. Animals were scored daily for 5–7 days following infection, and estrous stages were simultaneously tracked daily by microscopic examination of cells collected by vaginal lavage. The maximum CDI score for each mouse was determined and compared with its estrous stage 48 h prior to the maximum score. Animals that were at estrus (blue), metestrus (orange), diestrus (green), late diestrus/early proestrus (pink), proestrus (purple), and late proestrus/early estrus (yellow) at 48 h were pooled together. The number of animals in each cycle varies as each individual animal progresses through the estrous cycle. Each individual mouse is denoted by a circle. Horizontal solid lines represent median values, dotted lines represent interquartile ranges, and tapered ends represent maximum and minimum values. Single-factor ANOVA was performed to assess differences among the sign severity means of the different estrous stages. ANOVA results with *P* values of <0.05 were analyzed *post hoc* using the Scheffe test for pairwise comparison between all groups. Columns that are labeled with different letters are statistically different. Columns that are labeled with the same letter are statistically similar.

Since the maximum CDI signs for the second cohort did not develop until 2 days post-challenge, the temporal effect of the estrous stage was also delayed, less pronounced, and more heterogeneous (Fig. 5) compared with animals in the first cohort (Fig. 4B). Indeed, second cohort animals that were in the estrus stage showed mild CDI signs 2 days later. In contrast, second cohort animals in the proestrus stage developed more severe CDI signs 2 days later.

### Combining murine estrous stages across experiments reveals that higher CDI severity at 24 h post-infection is correlated with proestrus

To increase statistical power, mice from the previous two cohorts were binned together into four estrous stages (proestrus, estrus, metestrus, diestrus) and analyzed at both 24- and 48-h timepoints relative to max CDI scoring. Transitional estrous classifications (e.g., proestrus/estrus, late diestrus, and early estrus) were classified as ambiguous and handled as described in the methods. To ensure these classifications did not bias the analysis, results were evaluated both including all animals and after excluding ambiguous cases.

**Fig 6:**
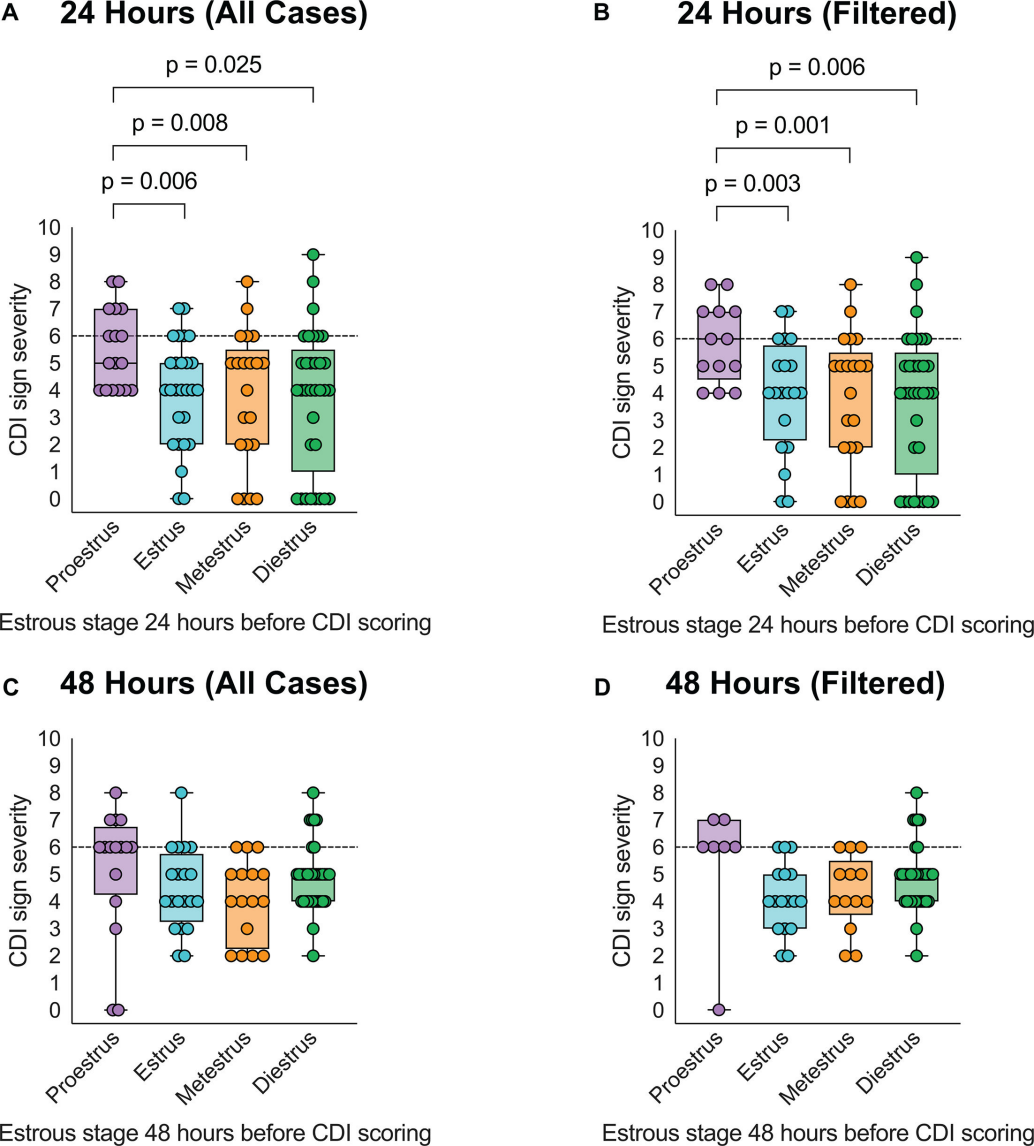
Analysis of combined mouse cohorts reveals a robust relationship between estrous stage and max CDI sign severity. Data from mice across two experiments were pooled and analyzed using a general linear model to determine whether the estrous stage affects CDI severity while accounting for differences between data sets. Proestrus/estrus, late diestrus, and early estrus stages were re-binned into the four primary stages (proestrus, estrus, metestrus, diestrus) using an indicator variable to identify ambiguous calls. Max CDI scores were plotted against estrous stage at (**A**) 24 h before scoring for all mice, (**B**) 24 h before scoring with ambiguous cases removed, (**C**) 48 h before scoring for all mice, and (**D**) 48 h before scoring with ambiguous cases removed. Non-significant comparisons (*P* > 0.05) are not shown. Pooling confirms that at 24 h before max CDI scores, the estrous stage appears to be a strong determinant of CDI severity. Notably, animals in proestrus 24 h before scoring have statistically higher CDI than all other stages. This effect is further amplified with ambiguous cases removed. However, this relationship appears to dissipate with estrous stages 48 h prior to max CDI scores, which are not presenting as statistically significant.

At 24 h, the estrous stage had a significant effect on CDI severity (F(3,94) = 4.759, *P* = 0.004), with *post hoc* comparisons showing higher CDI severity in proestrus compared with estrus (*P* = 0.006), metestrus (*P* = 0.008), and diestrus (*P* = 0.025) (Fig. 6A). This pattern was reinforced when ambiguous estrous stage classifications were removed (Fig. 6B), with proestrus again showing significantly higher CDI severity compared with estrus (*P* = 0.003), metestrus (*P* = 0.001), and diestrus (*P* = 0.006), indicating that the observed effect is not driven by uncertainty in staging. Batch effects were also significant at 24 h (all cases: F(1,94) = 13.957, *P* < 0.001; ambiguous removed: F(1,72) = 15.954, *P* < 0.001).

In contrast, no significant differences in CDI severity were observed between estrous stages at 48 h post-infection when all animals were included (Fig. 6C), despite trends observed in individual cohort analyses (Fig. 5), likely reflecting increased variability and reduced statistical power when cohorts are analyzed separately. This remained unchanged after removal of ambiguous classifications (Fig. 6D), indicating that the lack of significance is not driven by staging uncertainty. By combining cohorts and accounting for batch effects, these apparent differences were not maintained as statistically significant. Although variation in CDI scores between groups was observed, these differences were not statistically significant, indicating that the influence of estrous stage on CDI severity is most pronounced early in infection and diminishes over time.

### Biomarkers obtained before infection (D0) and during early infection (D1) correlate with CDI progression

Pre-infection blood samples (D0, obtained hours before spore challenge) and early infection blood samples (D1, 1 day after spore challenge) were obtained from female mice to test for the effect of hormones, cytokines, and immunoglobulins on CDI progression (Tables S2 to S11).

To evaluate relationships between circulating biomarkers and CDI severity, we performed pairwise correlation analyses across all measured variables. Correlations were calculated using Kendall’s Tau Coefficient, a non-parametric method that assesses the strength and direction of associations without assuming normal distributions. Positive correlations indicate that higher levels of one variable are associated with higher levels of another, while negative correlations indicate inverse relationships. Correlation strength was interpreted based on the magnitude of the coefficient, and statistical significance was defined at *P* < 0.10.

**Fig 7:**
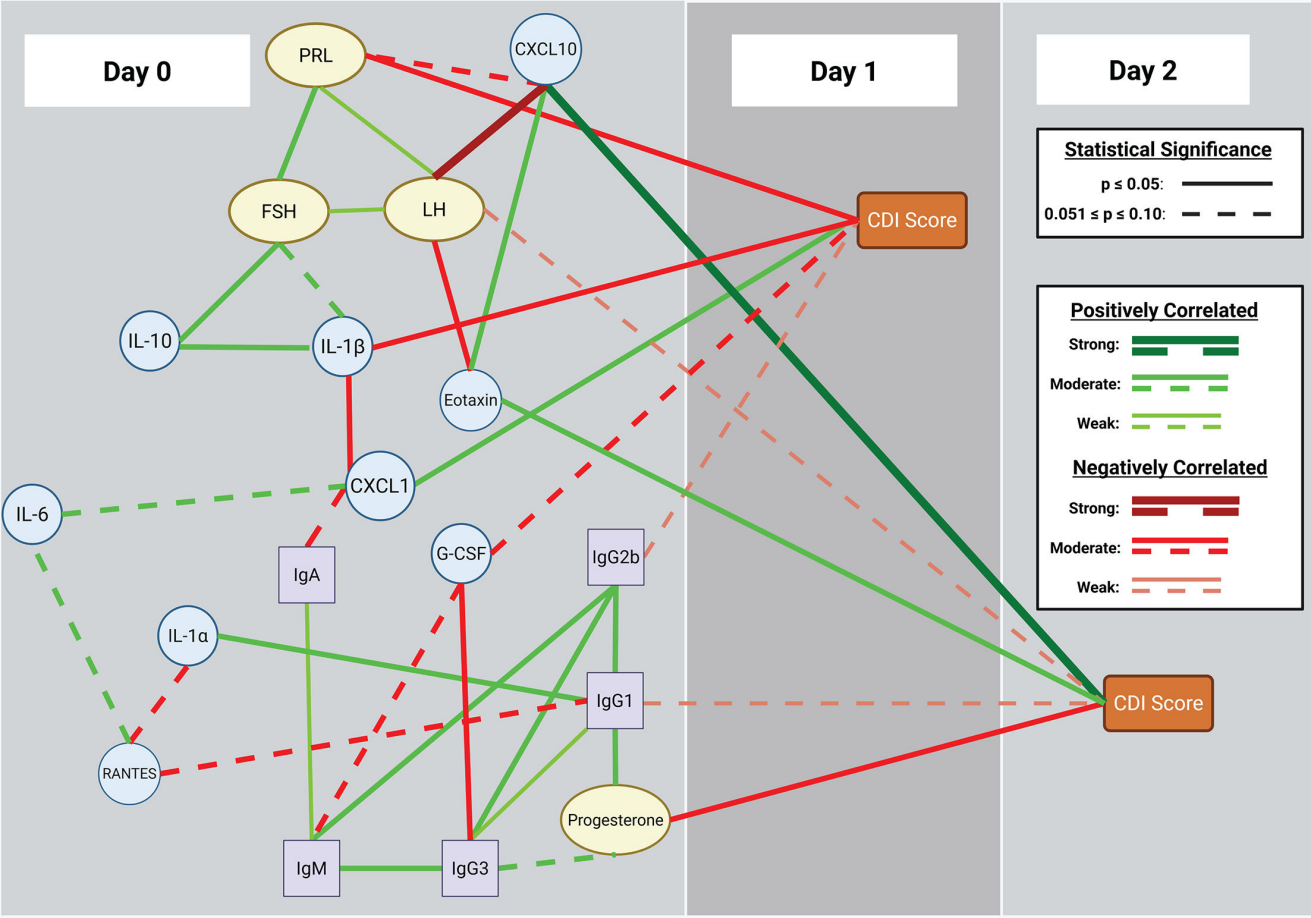
Biomarker networks before infection. Serum was obtained from female mice before challenge (D0) with *C. difficile* spores and individually analyzed for concentrations of sex hormones, cytokines, and immunoglobulins. Animals were scored for CDI sign severity on days 1–5 post-challenge. Days with correlations between biomarkers and CDI severity are highlighted (days 1 and 2 post-challenge). Biomarker levels and CDI scores were correlated using Kendall-tau. Positive correlations are represented with green lines. Negative correlations are represented with red lines. Line thickness and hues represent the strengths of the correlations. Solid lines represent statistically significant correlations with *P* ≤ 0.05. Dashed lines represent statistically significant correlations with 0.051 < *P* ≤ 0.10.

Correlation analysis of pre-infection (D0) biomarkers forms two, almost separate clusters. In fact, pro-inflammatory chemokine KC (CXCL1) is the only connection between the two clusters. Cluster 1 (Fig. 7, top) is centered around pituitary hormones FSH, PRL, and LH. Cluster 2 (Fig. 7, bottom), on the other hand, is centered around the steroidal hormone progesterone.

In cluster 1, pre-infection (D0) levels of hormone LH, hormone FSH, and anti-inflammatory cytokine IL-10 form a positively correlated network that negatively influences CDI severity during the following day (day 1 after spore challenge) through PRL and pro-inflammatory cytokine IL-1β. Only chemokine KC (CXL1) positively correlated with CDI sign on day 1 post-challenge, while simultaneously negatively correlating with IL-1β and IgA levels and positively correlating with pleiotropic cytokine IL-6.

In cluster 2, pre-infection (D0) levels of hormone progesterone, pro-inflammatory cytokine IL-1α, immunoglobulin IgG1, immunoglobulin IgG3, and immunoglobulin IgM form a complex positively correlated group that negatively affects CDI severity on the following day through immunoglobulin IgG2b. From the same group, IgM and IgG3 negatively correlate with anti-inflammatory cytokine G-CSF. In turn, G-CSF negatively influences CDI score on day 1.

Mirroring the temporal delay between estrous stages and CDI severity, pre-infection (D0) levels of PRL, FSH, LH, pro-inflammatory cytokines IP-10 (CXCL10), and eotaxin form a group that positively correlates with CDI severity 2 days post-challenge. The same network negatively affects CDI severity 2 days post-challenge through LH. The 2-day delayed effect is also observed in cluster 2. Pre-infection (D0) levels of the progesterone/immunoglobulin group negatively influence CDI severity 2 days post-challenge through IgG1 and progesterone.

**Fig 8:**
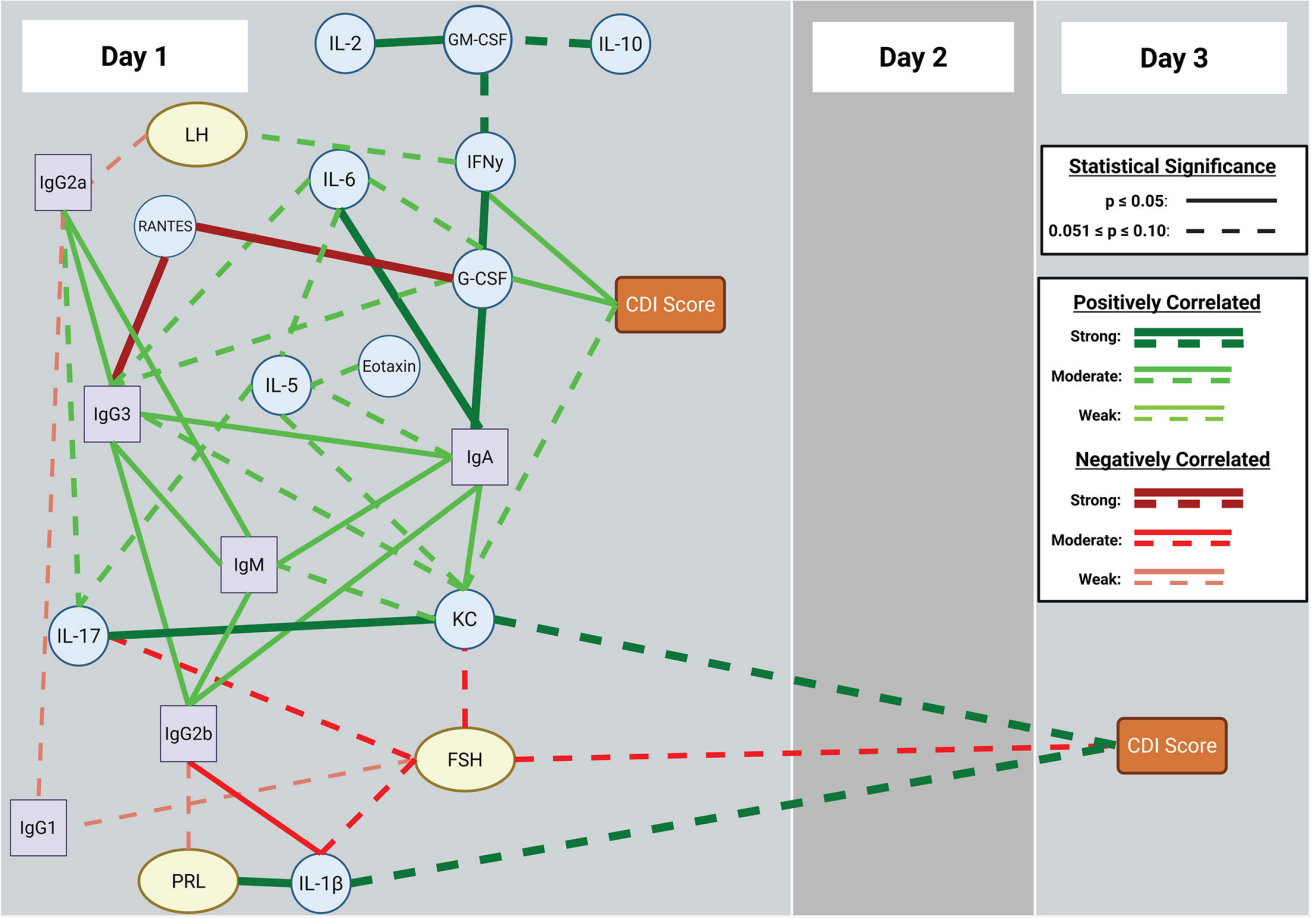
Biomarker networks during early infection. Serum was obtained from female mice the day after challenge (D1) with *C. difficile* spores and individually analyzed for concentrations of sex hormones, cytokines, and immunoglobulins. Animals were scored for CDI sign severity on days 1–5 post-challenge. Days with correlations between biomarkers and CDI severity are highlighted (days 1 and 3 post-challenge). Biomarker levels and CDI scores were correlated using Kendall-tau approaches. Positive correlations are represented with green lines. Negative correlations are represented with red lines. Line thickness and hues represent the strengths of the correlations. Solid lines represent statistically significant correlations with *P* ≤ 0.05. Dashed lines represent statistically significant correlations with 0.051 < *P* ≤ 0.10.

As expected after spore challenge, early infection (D1) immunoglobulin, cytokine, and chemokine levels create a single complex network that correlates CDI severity on the same day (Fig. 8. Early infection levels of LH and FSH positively influence same-day CDI sign severity through pro-inflammatory cytokines IFNγ and KC, respectively.

We also observed a temporal effect between early infection biomarkers and CDI recovery, 3 days post-challenge. Indeed, early infection (D1) levels of KC and IL-1β show a strong positive correlation to CDI severity on day 3 post-challenge. Central to these effects, early infection (D1) FSH levels show a strong negative correlation with early infection (D1) levels of KC and IL-1β. FSH also shows a negative correlation with CDI severity on day 3 post-challenge.

## DISCUSSION AND CONCLUSIONS

Few attempts have been made to understand how sex affects CDI onset and severity. To evaluate the clinical significance of sex in human CDI, we conducted an extensive PUBMED search for primary articles related to CDI risks. We found that most studies (*n* = 87) did not stratify results by sex. Of the studies that considered sex as a variable (*n* = 24), most linked CDI to other comorbidities. These studies often had small sample sizes, making it hard to determine whether sex was directly associated with CDI or with the underlying comorbidities. However, when CDI was the primary diagnosis—and after adjusting for other risk factors—several large cohort studies demonstrated that women are at a higher risk than men of developing symptoms (7, 8, 10, 11, 44). In fact, a multicenter retrospective cohort study involving over 1 million patients across 150 U.S. hospitals found that women had 1.2 times the odds of developing CDI compared with men (44).

These studies are complicated by the effect of age as a risk factor for CDI. Indeed, the incidence of CDI remains steady for younger populations and shows a sharp increase for both men and women over 50 years old (45). For many patients, this would be consistent with the onset of menopause in women and andropause in men. Unfortunately, these studies did not segregate their data based on these potential hormonal changes.

Although the correlation between sex and human CDI is clear, testing for susceptibility in human females is cost-prohibitive and impractical. In this study, the murine model recapitulates many of the differences in CDI outcome observed between sexes in humans (7, 8, 10, 11, 46).

When animals were infected with any of the *C. difficile* strains tested, female mice developed statistically more severe CDI than their male counterparts. The timing of these differences varied, but in every case, the most significant differences (*P* < 0.1) in CDI severity coincided with the peak of disease severity in their respective infection timeline. Because animals challenged with the hypervirulent *C. difficile* strain R20291 showed the strongest CDI signs, we continued to use this strain for all subsequent experiments.

In contrast to hamsters, murine CDI seldom tends to be lethal (34). However, when animals were fed a diet known to exacerbate CDI signs, the mortality of male mice increased significantly. Even then, male mice showed less severe signs than females fed the same diet. This shows that the sexual dimorphism of murine CDI is still present under conditions where CDI is severe.

The most obvious difference between male and female mice is the female estrous cycle. Indeed, we found that estrous stages have a 1-day to 2-day delayed effect on CDI sign severity. The delayed effect of the estrous stage on CDI severity might be due to the time needed for sex hormones to affect host physiological responses (e.g.*,* immune system, intestinal microbiota, steroidal hormone accumulation in the gut) during infection.

Little is known regarding the role of sex hormones in intestinal infection, and their effect on adaptation of the immune response in the GI tract. It should be noted that male and female mice may have distinct microbiota, particularly when they are not co-housed. Male mice may harbor more protective microbiota, which could potentially influence CDI. However, a study by Wallace et al. found that murine female intestinal microbiota does not appear to shift through the estrous cycle (47).

The interplay between estrous stage cycling and CDI progression is quite complex. For CDI caused by *C. difficile* strain R20291, infection starts and reaches a maximum of severity on days one or two following spore challenge. Surviving animals completely recover by days 4–5 post-challenge. In contrast, the estrous cycle can take between 3 and 6 days to be completed. Furthermore, the duration of each estrous stage is variable, with proestrus and metestrus lasting as little as 8 h and diestrus lasting as long as 72 h (48). This was further complicated by the observation that estrous stages showed a 1–2-day delayed effect on CDI severity. Thus, binning animals into different estrous stages for each day reduced our statistical power for some of the estrous stages. Nevertheless, our data clearly show that animals challenged while in the estrus stage were largely protected from CDI. In contrast, animals challenged while in the proestrus stage developed deadly CDI.

To address this variability and improve our ability to detect meaningful patterns, we combined animals across experiments and binned them by estrous stage at defined timepoints. Because experiments were conducted across independent cohorts, this approach also allowed us to ensure that differences in CDI severity were not driven by variability between experiments.

This approach increased the number of observations within each stage, particularly for shorter stages such as proestrus, which are otherwise underrepresented when analyzed within individual experiments or days. Importantly, this approach allowed us to capture consistent trends that were not always apparent when analyzing smaller subsets of data, including the clear increase in CDI severity associated with proestrus at earlier timepoints. Additionally, performing analysis both with and without ambiguous estrous classifications confirmed that these patterns were robust and not driven by staging uncertainty. Together, this supports the conclusion that binning across experiments provides a more reliable representation of the relationship between estrous stage and CDI severity.

As expected, pre-infection (D0) hormone levels varied between individuals since animals were at different estrous stages when they were challenged with *C. difficile* spores on day 0. Interestingly, we also found inter-individual variabilities for pre-infection levels of cytokines and immunoglobulins, even though blood was collected before spore challenge. In contrast, early infection (D1) cytokine and immunoglobulin levels were more varied, as expected for animals progressing through active infection and showing different sign severities.

The effect of pre-infection immune biomarkers on CDI onset and severity is quite complex, since anti-inflammatory, pro-inflammatory, and pleiotropic cytokines correlate with sign severity days later. Nevertheless, we observe feedback loops for cytokines that directly correlate with CDI severity and are only weakly hormone-dependent. For example, both KC (CXL1) and IL-1β are pro-inflammatory cytokines. However, high levels of pre-infection KC (CXL1) lead to more severe CDI on day 1 after spore challenge. In contrast, high levels of IL-1β were protective. Furthermore, KC (CXL1) and IL-1β are negatively correlated. Hence, we inferred that the balance of pre-infection levels of KC (CXL1) and IL-1β would be a primary determinant for the outcome of CDI early in the infection process.

In contrast, high pre-infection levels of pro-inflammatory cytokines IP-10 (CXCL10) and eotaxin lead to more severe CDI signs, but the effect happens two days after challenge. The pre-infection levels of both IP-10 (CXCL10) and eotaxin are negatively correlated with gonadotropic hormones, especially LH. Although LH is mostly regarded as a reproductive hormone, studies have shown that LH can slow gastric motility (49). To our knowledge, the role of LH in intestinal infections has not been reported.

IP-10 (CXCL10) has been reported as a potential biomarker for human CDI severity (50). Eotaxin is a chemokine that recruits eosinophils to areas of inflammation and has been shown to play a role in the onset of the murine estrus cycle (44). Similar to IP-10 (CXCL10), higher eotaxin levels have been associated with more severe CDI in humans (51). Eotaxin eosinophil recruitment has also been shown to be necessary to prevent severe tissue damage in CDI (51). Hence, we speculate that pre-infection gonadotropic hormone spikes during the murine protective-estrus stage may cause a decrease in pre-infection IP-10 (CXCL10) production, resulting in less severe disease.

PRL is both a hormone and a cytokine with roles in reproduction, lactation, and stress regulation (52). The negative correlation of pre-infection PRL with early CDI signs mirrors the protective effect of the estrus cycle. This is consistent with the typical chronological presentation of hormones in the murine estrous cycle, with PRL levels rising approximately 1 day (estrus stage) before the elevation of progesterone levels (metestrus-diestrus stage). However, pre-infection PRL does not seem to directly correlate with any cytokine, chemokine, or immunoglobulin that affects early CDI progression. Thus, the mechanism linking PRL levels to CDI onset is unclear.

Similar to LH, we found a negative correlation between pre-infection progesterone levels and CDI severity 2 days later. During pregnancy in humans and mice, progesterone stimulates the expression of IL-4 and IL-10 and favors the activation of Th2 cytokines while suppressing the major Th1 cytokines, IL-12p70, IL-2, IFN-γ, and IL-18 (53–55). However, in our study, progesterone did not show any direct relationships with any chemokines and cytokines.

In contrast to the lack of effect on cytokines, we observed a positive correlation network between pre-infection progesterone levels and pre-infection IgG subtypes, that seems to protect mice from CDI. IgG1 and IgG2 are the most abundant circulating immunoglobulin G subclasses. While IgG1s respond to foreign peptides and are effective in opsonization, IG2s respond to bacterial capsular polysaccharides (56). Interestingly, murine, human, and nonhuman primate cervicovaginal IgG subtypes are highest during times of increased progesterone (57). How progesterone might affect pre-infection immunoglobulin levels to help ameliorate CDI signs is not currently known. However, a recent study showed that exposure to progesterone resulted in decreased *Listeria monocytogenes* burden in cultured intestinal cells (58).

We have previously shown that progesterone can inhibit *C. difficile* spore germination *in vitro* (32). Interestingly, sex steroidal hormones are also present at low concentrations in the intestinal tract and show a 1-day accumulation delay compared with serum (59), a timeline reminiscent of the delayed effect of progesterone on murine CDI. Due to the heterogeneous environment of the intestinal lumen, there could be localized areas of higher hormone concentrations where they could interact with *C. difficile* spores. Whether progesterone affects CDI indirectly by modulating the immune system or directly by inhibiting spore germination needs to be elucidated.

Pre-infection IgG1 and IgG3 also showed correlations with cytokines and chemokines. IgG1 positively correlated with IL-1α, a dual role cytokine involved in homeostasis and the immune response (60). In turn, IgG1 and IL-1α negatively correlated with proinflammatory cytokine RANTES. On the other hand, IgG3 negatively correlated with the proinflammatory cytokine G-CSF.

Stimulated macrophages and other immune cells produce G-CSF and RANTES in response to infection. Both are important as chemoattractants and aid in the maturation and activation of immune cells (61, 62). In fact, *C. difficile* has been shown to initiate a strong G-CSF and RANTES expression in the mouse intestinal tract, which may be used as markers for the severity of disease (63, 64). The direct and indirect effects of progesterone on immunoglobulins and cytokines could account for protection from CDI in mice.

The fact that pre-infection (D0) levels of progesterone and LH show a time-delayed, negative correlation to CDI severity seems counterintuitive, given the deathly effect of proestrus on CDI. It should be noted that the boundaries between estrous stages are arbitrary and dependent on cellular response to hormonal changes. In contrast, changes in sex hormone levels follow a time continuum throughout the estrous cycle. For example, even though progesterone is expected to fall during estrus, entry into the stage happens when progesterone levels are at their maximum. Hence, small variations in sample collection timing could result in uncoupling between expected sex hormone levels and identified estrous stages. Indeed, sex hormone fluctuations have been shown to follow mice strain-specific patterns (65) and even show significant variabilities between individuals on the same assigned estrous stages. These temporal uncertainties could partially explain the paradoxical nature of sex hormone effects on CDI severity.

After infection onset, the effect of sex hormones on disease progression seems to weaken. This might be due to the complex immune response that occurs after infection onset. At this point, the robust feedback loop of the immune system would become more important than the more subtle effects of sex hormones.

Nevertheless, we found a potential PRL/FSH effect on CDI recovery. Early infection PRL levels positively correlated with IL-1β. Meanwhile, early infection FSH negatively correlated with both IL-1β and KC. In a mutual feedback loop, IL-1β can stimulate the secretion of PRL, and PRL can increase the secretion of IL-1β through stimulated differentiation of T cells (52, 66). Although IL-1β is typically considered a pro-inflammatory signal stimulated by infection, IL-1β activity is complex and also plays a role in commensal microbiota regulation and maintenance (67). We are likely seeing this early infection loop playing a role in CDI recovery.

Little is known regarding the effect of FSH on cytokine and chemokine production in the intestine of humans or mice. In fact, receptors for FSH are not highly expressed in the intestine (68). In osteoclast differentiation, FSH has also been shown to induce the expression of IL-1β and IL-6 from monocytes (69, 70). Hence, in the murine intestine, both pre-infection and early infection FSH may have a role in the upregulation of IL-1β and IL-10 to aid in tissue maintenance and repair.

We have previously shown that *C. difficile* spores germinate between 6- and 9-h post-challenge in the intestines of mice (36). Potentially, the strong association between day 0 estrous stages, pre-infection biomarker levels, and CDI severity on days 1–2 could be due to estrous cycle and hormone fluctuations influencing the initial stages of CDI onset. On the other hand, more subtle delayed effects of the estrous cycle and sex hormones seem to continue throughout CDI progression into infection recovery. This suggests that estrous stages can increase or decrease female mice’s susceptibility to CDI both at the early and late stages of the infection course.

The full host immune response initiated by infection with *C. difficile* spores is yet to be fully elucidated. Many reports have identified cytokine and chemokine correlations between expression and severity of disease, with similarities and differences in these reports, suggesting dual roles in pathogenicity and protection (71–75). There is likely a very complex crosstalk between the systemic host immune response, the local immune cells, and intestinal microbiota, resulting in a fine line between infection clearance and tissue damage.

The primary goal of this study was to determine whether the estrous cycle, and thus hormone status, plays a role in modulating CDI in mice. Therefore, serum immune markers and hormone levels were prioritized in these large cohorts of mice. Now that the estrous cycle has been shown to correlate with CDI, bacterial burdens and *C. difficile* toxin levels can be examined in future studies to determine the effects of hormones, such as progesterone, on bacterial colonization/load, spore germination, and toxin accumulation *in vivo*.

Approaching *C. difficile* infection by studying the hypothalamic-pituitary-gonadal (HPG) axis allows us to determine how sex hormones and the estrus cycle affect the severity and progression of CDI. Many cells of the immune system contain receptors for sex hormones, which can activate cytokines, showing a connection between immune modulation and sex hormones. Although the results obtained in this work are tantalizing, we mostly relied on correlation studies. Although less likely than the direct effects of hormone levels on the host immune response, we cannot rule out the potential effect of hormone-mediated intestinal microbiota changes on CDI severity. Similarly, it is possible that at least progesterone could directly affect *C. difficile* spore germination and thus prevent CDI onset. Hence, the exact mechanism linking hormonal changes to CDI severity still needs to be elucidated. The effect of individual hormones on CDI severity needs to be established but provides an open door into explorations on the relationship between sex hormones, the gut, and CDI.

## SUPPLEMENTAL MATERIAL

Supplemental material iai.00243-26-s0001.pdf Fig. S1 and S2; Tables S1 to S11.

ASM does not own the copyrights to Supplemental Material that may be linked to, or accessed through, an article. The authors have granted ASM a non-exclusive, world-wide license to publish the Supplemental Material files. Please contact the corresponding author directly for reuse.
