# Supplementary material for: Effects of sexual dimorphism and estrous cycle on murine Clostridioides difficile infection
Source: Infect Immun. Author manuscript; Available in PMC 2026 Aug 21. (PMC13460132; doi:10.1128/iai.00243-26)
Supplement: Supplementary file 1 [file NIHMS2200852-supplement-Supplementary_materials.pdf]

Supplementary Materials

Supplementary figures

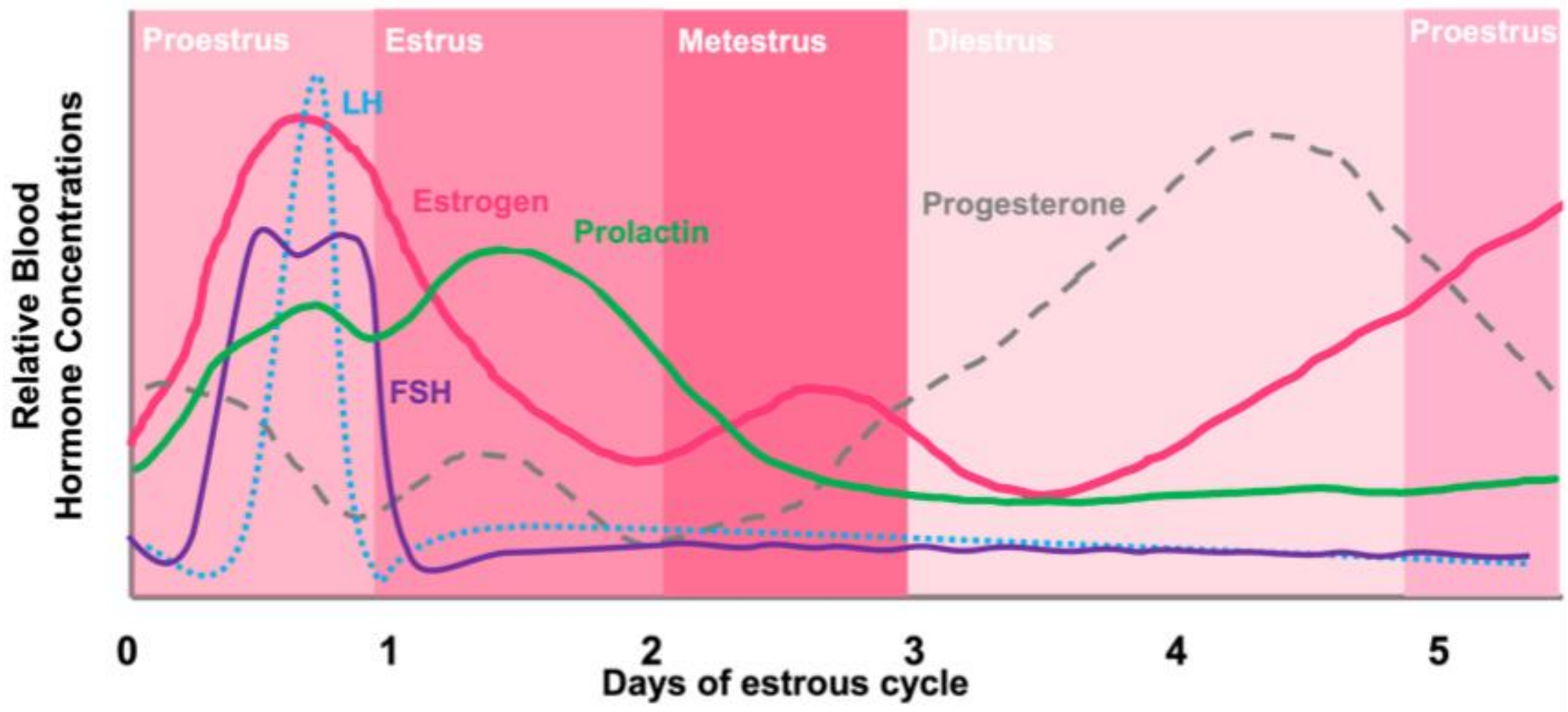

Figure S1. *A generalized depiction of the relative concentrations of sex-associated hormones during each stage of the estrous cycle adapted from McLean et al., 2012.* Proestrus is associated with elevated levels of several sex-associated hormones including follicle stimulating hormone (FSH), estrogen, and prolactin, followed by a sharp spike in luteinizing hormone (LH). Between estrus and metestrus is a steady decrease in those hormones. Towards the end of metestrus into the later part of diestrus is a gradual increase in the sex steroid progesterone which begin to drop toward the end of diestrus before the estrous cycle restarts.

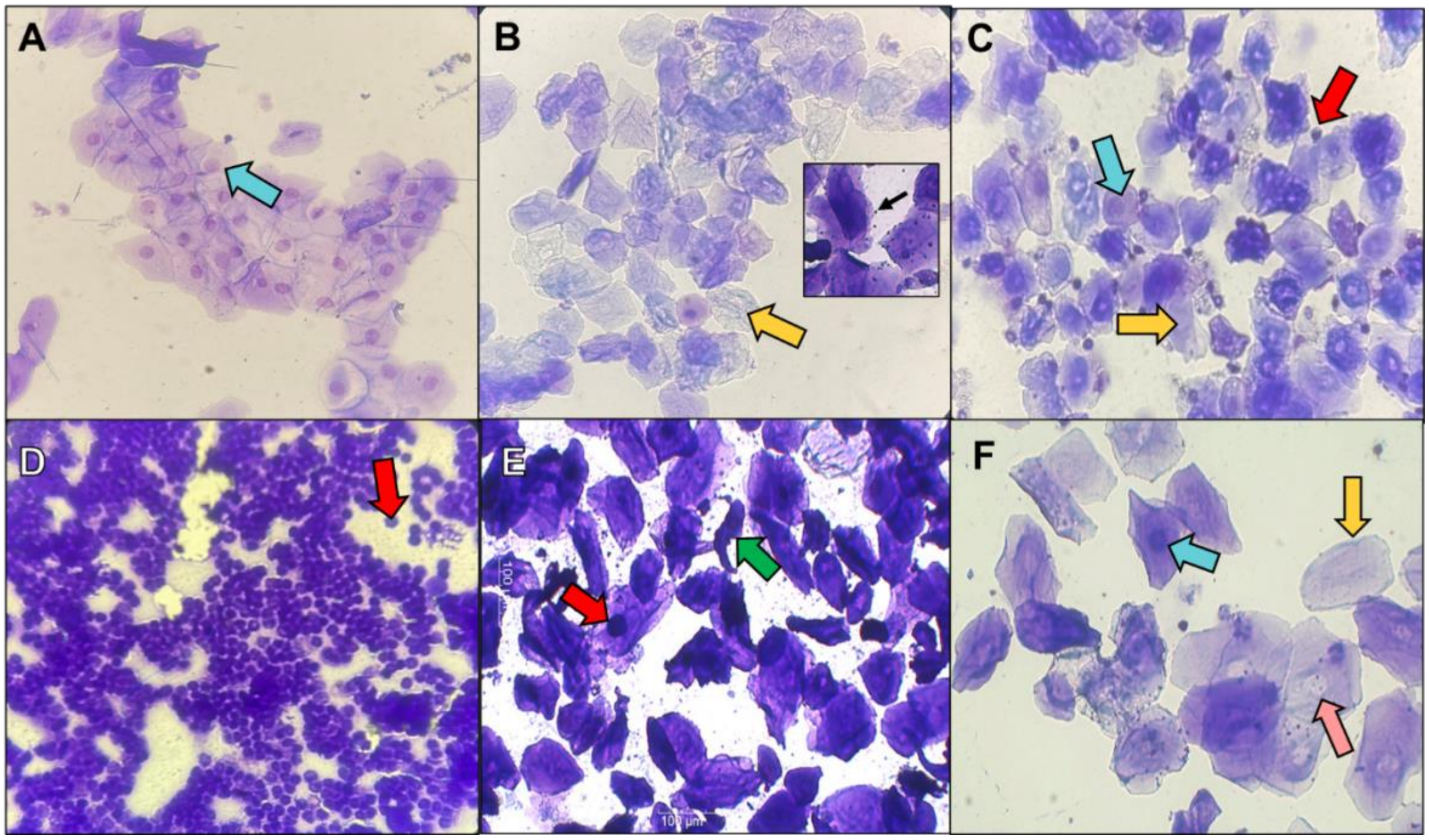

Figure S2. *Estrous stages determination by vaginal cytology.* Vaginal swabs were taken and spread on microscopy glass slides. After air-drying, slides were stained with crystal violet. Estrous cycle stages can be identified based on the presence of either nucleated epithelial cells (blue arrows), cornified (keratinized) anucleated epithelial cells (yellow arrows), leukocytes (red arrows), or combinations of these cell types. (A) proestrus stage is dominated by the presence of nucleated epithelial cells. (B) When animals are in the estrus stage, cornified anucleated epithelial cells are in abundance. This stage can also show the presence of small specks of bacteria (black arrow in insert) adhering to the epithelial cells. (C) In the metestrus stage, all three major cell types are present. (D) When animals transition to the diestrus stage, samples contain mostly leukocytes with scattered appearances of a few nucleated epithelial cells. Cytologic characteristics can also show intermediate stages of the estrous cycle. (E) Small, irregular shaped cells (green arrow) can signify the formation of nucleated cells and are representative of late diestrus/early proestrus. Some lingering leukocytes may also be present at

this stage. (F) Late proestrus/early estrus is usually marked by a transition of nucleated cells to enucleation leaving a halo-like appearance in the middle of the cell (pink arrow).

Supplementary tables

Table S1. *Clostridioides difficile* strains used in this study			
**Strain**	**Obtained from**	**Origin**	**Source**
630	ATCC	Laboratory strain	ATCC #BAA-1382
R20291	Nigel Minton Lab	The Netherlands	Ed Kujiper
9001966	Nigel Minton Lab	Stoke Madeville, UK	Ed Kujiper

The complete set of correlation analyses is shown in Tables S2-S11. Positive correlations are highlighted in green. Negative correlations are highlighted in red. Dark colors represent strong correlations. Medium colors represent moderate correlations. Light colors represent weak correlations. Lack of correlations are shown as non-highlights.

Table S2. Murine CDI severity correlate with murine CDI severity			
**Day of CDI Scoring**	**Day of Bleed**	**Day of CDI Scoring**	**Kendall's Tau Analysis**
5	0	4	τ(16)=0.74, p=0.001
5	1	2	τ(10)=0.7, p=0.03
4	1	3	τ(10)=0.58, p=0.05
4	0	3	τ(17)=0.54, p=0.01
5	0	3	τ(16)=0.49, p=0.03
4	0	1	τ(17)=0.35, p=0.1
4	1	2	τ(10)=0.39, p=0.18
5	1	4	τ(10)=0.39, p=0.21
2	1	1	τ(15)=0.34, p=0.13
4	0	2	τ(17)=0.31, p=0.137

3	0	1	τ(17)=0.24,<br>p=0.28
5	0	2	τ(16)=0.23,<br>p=0.31
3	0	2	τ(17)=0.22,<br>p=0.31
3	1	1	τ(10)=0.2,<br>p=0.51
5	0	1	τ(16)=0.18,<br>p=0.44
2	0	1	τ(19)=0.16,<br>p=0.42
4	1	1	τ(10)=0.16,<br>p=0.58
5	1	3	τ(10)=0.13,<br>p=0.68
3	1	2	τ(10)=0.09,<br>p=0.76
5	1	1	τ(10)=0,<br>p=1

Table S3. Hormone levels correlate with murine CDI severity			
**Hormone**	**Day of Bleed**	**Day of CDI Scoring**	**Kendall's Tau Analysis**
LH	1	4	τ(10)=0.42, p=0.12
PRL	1	5	τ(10)=0.37, p=0.19
PRL	1	4	τ(10)=0.34, p=0.21
PRL	1	1	τ(16)=0.18, p=0.38
PRL	1	2	τ(15)=0.18, p=0.39
LH	1	1	τ(16)=0.16, p=0.44
LH	1	3	τ(10)=0.13, p=0.63
Progesterone	1	5	τ(8)=0.07, p=0.83
FSH	0	3	τ(17)=0.06, p=0.76
Progesterone	1	1	τ(12)=0.04, p=0.88
Progesterone	1	3	τ(8)=0.04, p=0.89
PRL	1	3	τ(10)=0.03, p=0.92
Progesterone	1	4	τ(8)=0, p=1
FSH	0	2	τ(19)=-0.01, p=0.94
Progesterone	0	3	τ(8)=-0.05, p=0.88
PRL	0	3	τ(17)=-0.08, p=0.69
LH	1	2	τ(15)=-0.09, p=0.65
Progesterone	0	5	τ(8)=-0.09, p=0.77
FSH	0	1	τ(22)=-0.14, p=0.41
LH	0	4	τ(17)=-0.14, p=0.47
Progesterone	1	2	τ(11)=-0.14, p=0.57

FSH	1	5	τ(10)=-0.15, p=0.6
FSH	0	4	τ(17)=-0.16, p=0.42
FSH	1	2	τ(15)=-0.16, p=0.45
LH	0	3	τ(17)=-0.23, p=0.25
FSH	0	5	τ(16)=-0.23, p=0.26
LH	0	5	τ(16)=-0.23, p=0.26
PRL	0	2	τ(19)=-0.24, p=0.2
FSH	1	1	τ(16)=-0.25, p=0.21
LH	1	5	τ(10)=-0.26, p=0.36
LH	0	1	τ(22)=-0.27, p=0.11
PRL	0	4	τ(17)=-0.28, p=0.15
PRL	0	5	τ(16)=-0.31, p=0.14
Progesterone	0	1	τ(11)=-0.32, p=0.23
FSH	1	4	τ(10)=-0.334, p=0.21
Progesterone	0	4	τ(8)=-0.37, p=0.23
LH	0	2	τ(19)=-0.33, p=0.08
PRL	0	1	τ(22)=-0.46, p=0.01
FSH	1	3	τ(10)=-0.49, p=0.07
Progesterone	0	2	τ(10)=-0.57, p=0.03

Table S4. Immunoglobulin levels correlate with murine CDI severity

Ig subtype	Day of Bleed	Day of CDI Scoring	Kendall's Tau Analysis
IgA	1	5	$\tau(10)=0.69$, $p=0.03$
IgA	1	4	$\tau(10)=0.48$, $p=0.1$
IgM	1	4	$\tau(10)=0.44$, $p=0.1$
IgG3	1	5	$\tau(10)=0.45$, $p=0.12$
IgA	1	3	$\tau(10)=0.42$, $p=0.15$
IgG3	1	4	$\tau(10)=0.39$, $p=0.15$
IgM	1	5	$\tau(10)=0.37$, $p=0.19$
IgM	1	3	$\tau(10)=0.34$, $p=0.21$
IgG2a	1	2	$\tau(15)=0.33$, $p=0.13$
IgG2a	1	5	$\tau(10)=0.31$, $p=0.34$
IgG2b	1	5	$\tau(10)=0.3$, $p=0.3$
IgG3	1	2	$\tau(15)=0.26$, $p=0.2$
IgM	0	3	$\tau(17)=0.24$, $p=0.22$
IgA	1	1	$\tau(16)=0.23$, $p=0.29$
IgA	0	4	$\tau(17)=0.22$, $p=0.26$
IgG2a	0	2	$\tau(19)=0.22$, $p=0.28$
IgG2a	1	1	$\tau(16)=0.22$, $p=0.3$
IgM	1	2	$\tau(15)=0.21$, $p=0.31$
IgA	0	3	$\tau(17)=0.19$, $p=0.35$
IgM	1	1	$\tau(16)=0.19$, $p=0.36$
IgG3	1	3	$\tau(10)=0.18$, $p=0.5$

IgG3	1	1	τ(16)=0.16, p=0.44
IgA	1	2	τ(15)=0.16, p=0.45
IgG1	0	3	τ(17)=0.15, p=0.45
IgG1	1	5	τ(10)=0.15, p=0.6
IgM	0	4	τ(17)=0.14, p=0.47
IgG2a	1	4	τ(10)=0.14, p=0.63
IgG2a	0	5	τ(16)=0.12, p=0.59
IgG3	0	3	τ(17)=0.1, p=0.63
IgG2b	1	1	τ(16)=0.08, p=0.7
IgG1	1	3	τ(10)=0.08, p=0.77
IgA	0	2	τ(19)=0.06, p=0.76
IgG2a	0	4	τ(17)=0.05, p=0.82
IgG2b	1	4	τ(10)=0.03, p=0.92
IgG2b	1	2	τ(15)=0.01, p=0.96
IgG2a	1	3	τ(10)=0, p=1
IgM	0	2	τ(19)=-0.03, p=0.88
IgG3	0	5	τ(16)=-0.03, p=0.88
IgM	0	5	τ(16)=-0.05, p=0.8
IgG3	0	4	τ(17)=-0.07, p=0.73
IgA	0	5	τ(16)=-0.07, p=0.76
IgG2b	1	3	τ(10)=-0.08, p=0.77
IgG3	0	2	τ(19)=-0.09, p=0.63
IgG1	0	1	τ(22)=-0.11, p=0.5

IgG2b	0	3	τ(17)=-0.11, p=0.57
IgG2a	0	1	τ(22)=-0.13, p=0.49
IgG1	0	4	τ(17)=-0.13, p=0.5
IgG1	1	1	τ(16)=-0.14, p=0.5
IgG3	0	1	τ(22)=-0.15, p=0.39
IgA	0	1	τ(22)=-0.15, p=0.39
IgG2a	0	3	τ(17)=-0.15, p=0.47
IgG1	1	4	τ(10)=-0.18, p=0.5
IgM	0	1	τ(22)=-0.22, p=0.19
IgG2b	0	4	τ(17)=-0.23, p=0.23
IgG2b	0	2	τ(19)=-0.24, p=0.2
IgG1	0	5	τ(16)=-0.24, p=0.24
IgG1	1	2	τ(15)=-0.26, p=0.2
IgG2b	0	5	τ(16)=-0.33, p=0.12
IgG2b	0	1	τ(22)=-0.33, p=0.05
IgG1	0	2	τ(19)=-0.33, p=0.07

Table S5. Hormone levels correlate with hormone levels

Hormone	Day of Bleed	Hormone	Kendall's Tau Analysis
PRL	0	FSH	$\tau(22)=0.45$, $p=0.003$
LH	0	PRL	$\tau(22)=0.34$, $p=0.03$
LH	0	FSH	$\tau(22)=0.31$, $p=0.05$
LH	1	PRL	$\tau(16)=0.15$, $p=0.42$
Progesterone	1	PRL	$\tau(12)=0.15$, $p=0.49$
Progesterone	1	FSH	$\tau(12)=0$, $p=1$
Progesterone	1	LH	$\tau(12)=0$, $p=1$
PRL	1	FSH	$\tau(16)=-0.07$, $p=0.72$
LH	1	FSH	$\tau(16)=-0.1$, $p=0.59$
Progesterone	0	LH	$\tau(10)=-0.11$, $p=0.65$
Progesterone	0	PRL	$\tau(10)=-0.14$, $p=0.59$
Progesterone	0	FSH	$\tau(10)=-0.23$, $p=0.37$

Table S6. Immunoglobulin levels correlate with immunoglobulin levels

Ig subtype	Day of Bleed	Ig subtype	Kendall's Tau Analysis
IgG3	0	IgM	$\tau(22)=0.68$, $p=0$
IgM	1	IgG3	$\tau(16)=0.67$, $p=0$
IgG3	1	IgA	$\tau(16)=0.54$, $p=0.006$
IgG3	1	IgG2a	$\tau(16)=0.51$, $p=0.01$
IgG3	0	IgG2b	$\tau(22)=0.5$, $p=0.001$
IgM	1	IgA	$\tau(16)=0.48$, $p=0.01$
IgG2b	0	IgG1	$\tau(22)=0.47$, $p=0.002$
IgM	1	IgG2a	$\tau(16)=0.47$, $p=0.02$
IgG2b	1	IgA	$\tau(16)=0.46$, $p=0.02$
IgM	0	IgG2b	$\tau(22)=0.42$, $p=0.006$
IgM	1	IgG2b	$\tau(16)=0.42$, $p=0.02$
IgG3	1	IgG2b	$\tau(16)=0.4$, $p=0.03$
IgM	0	IgA	$\tau(22)=0.32$, $p=0.05$
IgG3	0	IgG1	$\tau(22)=0.31$, $p=0.05$
IgG2b	1	IgG1	$\tau(16)=0.29$, $p=0.12$
IgG2a	1	IgA	$\tau(16)=0.29$, $p=0.17$
IgG2b	1	IgG2a	$\tau(16)=0.28$, $p=0.16$
IgG3	0	IgG2a	$\tau(22)=0.26$, $p=0.13$
IgM	0	IgG2a	$\tau(22)=0.25$, $p=0.14$
IgG2a	0	IgA	$\tau(22)=0.25$, $p=0.17$
IgM	0	IgG1	$\tau(22)=0.24$, $p=0.11$

IgG2b	0	IgA	$\tau(22)=0.23$, $p=0.17$
IgG2b	0	IgG2a	$\tau(22)=0.21$, $p=0.23$
IgG1	0	IgA	$\tau(22)=0.18$, $p=0.27$
IgG3	0	IgA	$\tau(22)=0.18$, $p=0.27$
IgG1	1	IgA	$\tau(16)=0.03$, $p=0.89$
IgM	1	IgG1	$\tau(16)=-0.01$, $p=0.96$
IgG2a	0	IgG1	$\tau(22)=-0.15$, $p=0.39$
IgG3	1	IgG1	$\tau(16)=-0.18$, $p=0.34$
IgG2a	1	IgG1	$\tau(16)=-0.34$, $p=0.08$

Table S7. Immunoglobulin levels correlate with hormone levels			
**Ig subtype**	**Day of Bleed**	**Hormone**	**Kendall's Tau Analysis**
IgG1	0	Progesterone	τ(10)=0.58, p=0.02
IgG3	0	Progesterone	τ(10)=0.45, p=0.07
IgM	0	Progesterone	τ(10)=0.36, p=0.15
IgM	1	Progesterone	τ(12)=0.32, p=0.15
IgG2b	0	Progesterone	τ(10)=0.27, p=0.28
IGg2b	0	FSH	τ(22)=0.2, p=0.2
IgA	0	LH	τ(22)=0.16, p=0.32
IgG2a	1	Progesterone	τ(12)=0.16, p=0.51
IgG2a	1	FSH	τ(16)=0.15, p=0.44
IgG2b	0	PRL	τ(22)=0.12, p=0.43
IgM	0	PRL	τ(22)=0.12, p=0.43
IgA	1	Progesterone	τ(12)=0.11, p=0.64
IgA	0	FSH	τ(22)=0.1, p=0.54
IgA	1	PRL	τ(16)=0.1, p=0.6
IgM	0	FSH	τ(22)=0.09, p=0.55
IgA	1	LH	τ(16)=0.09, p=0.64
IgG1	1	Progesterone	τ(12)=0.09, p=0.68
IgG3	1	Progesterone	τ(12)=0.09, p=0.68
IgG2b	0	LH	τ(22)=0.08, p=0.59
IgM	0	LH	τ(22)=0.08, p=0.59
IgG2a	1	PRL	τ(16)=0.08, p=0.7

IgG3	0	FSH	τ(22)=0.07, p=0.65
IgG3	0	LH	τ(22)=0.06, p=0.69
IgG1	0	PRL	τ(22)=0.05, p=0.76
IgG3	0	PRL	τ(22)=0.03, p=0.84
IgM	1	PRL	τ(16)=0.03, p=0.89
IgG1	0	LH	τ(22)=-0.03, p=0.87
IgG1	0	FSH	τ(22)=-0.07, p=0.65
IgA	0	PRL	τ(22)=-0.07, p=0.68
IgA	0	Progesterone	τ(10)=-0.072, p=0.783
IgG2a	0	LH	τ(22)=-0.08, p=0.63
IgG3	1	PRL	τ(16)=-0.08, p=0.69
IgG3	1	FSH	τ(16)=-0.09, p=0.62
IgG3	1	LH	τ(16)=-0.09, p=0.62
IgM	1	LH	τ(16)=-0.09, p=0.62
IgG1	1	LH	τ(16)=-0.09, p=0.65
IgG2a	0	FSH	τ(22)=-0.1, p=0.57
IgG2b	1	FSH	τ(16)=-0.11, p=0.56
IgG2b	1	Progesterone	τ(12)=-0.11, p=0.63
IgG1	1	PRL	τ(16)=-0.12, p=0.53
IgG2b	1	LH	τ(16)=-0.13, p=0.5
IgM	1	FSH	τ(16)=-0.14, p=0.44
IgG2a	0	PRL	τ(22)=-0.21, p=0.21
IgG2a	0	Progesterone	τ(10)=-0.23, p=0.38

IgA	1	FSH	τ(16)=-0.29, p=0.14
IgG2a	1	LH	τ(16)=-0.33, p=0.10
IgG1	1	FSH	τ(16)=-0.35, p=0.06
IgG2b	1	PRL	τ(16)=-0.36, p=0.05

Table S8. Cytokine levels correlate with murine CDI severity

Cytokine	Day of Bleed	Day of CDI Scoring	Kendall's Tau Analysis
KC	1	3	τ(4)=0.913, p=0.071
IL-1b	1	3	τ(4)=0.913, p=0.071
IL-1b	1	4	τ(4)=0.913, p=0.071
IP-10	0	2	τ(9)=0.73, p=0.01
Eotaxin	0	5	τ(9)=0.72, p=0.01
G-CSF	1	1	τ(7)=0.688, p=0.039
Eotaxin	0	4	τ(9)=0.67, p=0.02
IFNy	1	1	τ(7)=0.658, p=0.072
KC	0	1	τ(10)=0.63, p=0.02
Eotaxin	0	2	τ(9)=0.61, p=0.03
IL-17	0	4	τ(9)=0.61, p=0.03
Eotaxin	0	3	τ(9)=0.59, p=0.04
KC	1	1	τ(7)=0.582, p=0.081
IL-10	0	5	τ(9)=0.56, p=0.09
IL-15	0	3	τ(9)=0.52, p=0.07
KC	1	5	τ(4)=0.707, p=0.18
IL-1b	1	5	τ(4)=0.707, p=0.18
IL-6	1	5	τ(4)=0.707, p=0.18
G-CSF	1	5	τ(4)=0.707, p=0.18
KC	1	4	τ(4)=0.548, p=0.279
IL-6	1	3	τ(4)=0.548, p=0.279

G-CSF	1	4	τ(4)=0.548, p=0.279
IFNy	0	2	τ(9)=0.52, p=0.11
IL-10	1	4	τ(4)=0.516, p=0.346
IL-2	1	4	τ(4)=0.516, p=0.346
IFNy	1	4	τ(4)=0.516, p=0.346
GM-CSF	1	4	τ(4)=0.516, p=0.346
IL-10	0	3	τ(9)=0.51, p=0.12
IL-4	0	5	τ(9)=0.49, p=0.15
TNFa	0	5	τ(9)=0.49, p=0.15
KC	1	2	τ(7)=0.488, p=0.129
IL-17	1	1	τ(7)=0.476, p=0.153
IP-10	0	5	τ(9)=0.46, p=0.11
IL-17	0	5	τ(9)=0.46, p=0.11
IL-4	0	4	τ(9)=0.45, p=0.16
TNFa	0	4	τ(9)=0.45, p=0.16
IL-1B	0	3	τ(9)=0.44, p=0.14
IP-10	0	4	τ(9)=0.43, p=0.13
IL-4	0	3	τ(9)=0.42, p=0.21
TNFa	0	3	τ(9)=0.42, p=0.21
IL-2	1	1	τ(7)=0.396, p=0.295
GM-CSF	1	1	τ(7)=0.396, p=0.295
IL-6	0	1	τ(10)=0.39, p=0.14
IL-1b	1	2	τ(7)=0.39, p=0.224

IL-17	1	2	τ(7)=0.39, p=0.224
IL-15	0	4	τ(9)=0.37, p=0.19
IL-6	1	1	τ(7)=0.37, p=0.266
RANTES	0	1	τ(10)=0.34, p=0.2
IL-15	0	1	τ(10)=0.34, p=0.2
IL-10	1	5	τ(4)=0.333, p=0.564
IL-2	1	5	τ(4)=0.333, p=0.564
IFNy	1	5	τ(4)=0.333, p=0.564
GM-CSF	1	5	τ(4)=0.333, p=0.564
IL-15	0	5	τ(9)=0.33, p=0.26
IL-10	0	4	τ(9)=0.33, p=0.3
RANTES	0	4	τ(9)=0.3, p=0.28
IL-2	1	2	τ(7)=0.274, p=0.449
GM-CSF	1	2	τ(7)=0.274, p=0.449
IL-10	1	2	τ(7)=0.27, p=0.443
IL-5	1	1	τ(7)=0.265, p=0.427
IL-17	0	3	τ(9)=0.26, p=0.37
IL-4	0	2	τ(9)=0.26, p=0.42
TNFa	0	2	τ(9)=0.26, p=0.42
IL-6	0	4	τ(9)=0.24, p=0.39
IL-10	0	2	τ(9)=0.24, p=0.45
IL-5	0	1	τ(10)=0.22, p=0.4
IL-17	0	1	τ(10)=0.22, p=0.4

IL-1b	0	5	τ(9)=0.21, p=0.49
IL-6	0	5	τ(9)=0.2, p=0.5
IP-10	0	3	τ(9)=0.2, p=0.5
G-CSF	1	2	τ(7)=0.195, p=0.543
IFNy	0	4	τ(9)=0.19, p=0.55
IL-5	1	3	τ(4)=0.183, p=0.718
IL-1a	1	3	τ(4)=0.183, p=0.718
IL-17	1	3	τ(4)=0.183, p=0.718
IL-6	1	4	τ(4)=0.183, p=0.718
G-CSF	1	3	τ(4)=0.183, p=0.718
IL-5	0	3	τ(9)=0.17, p=0.57
IL-1b	1	1	τ(7)=0.159, p=0.634
IFNy	0	5	τ(9)=0.14, p=0.68
IFNy	1	2	τ(7)=0.135, p=0.701
IL-1a	0	3	τ(9)=0.13, p=0.65
RANTES	0	5	τ(9)=0.13, p=0.65
RANTES	0	2	τ(9)=0.12, p=0.66
IL-15	0	2	τ(9)=0.12, p=0.66
IL-17	0	2	τ(9)=0.12, p=0.66
KC	0	4	τ(9)=0.12, p=0.67
IL-1a	0	1	τ(10)=0.1, p=0.71
IL-1b	0	2	τ(9)=0.1, p=0.74
IL-15	1	2	τ(7)=0.098, p=0.761

G-CSF	0	2	τ(9)=0.06, p=0.83
IL-1a	1	1	τ(7)=0.053, p=0.874
TNFa	0	1	τ(10)=0.05, p=0.86
IL-4	0	1	τ(10)=0.05, p=0.86
IL-5	0	2	τ(9)=-0.03, p=0.91
IL-5	0	4	τ(9)=-0.03, p=0.91
IP-10	0	1	τ(10)=-0.05, p=0.85
IL-2	0	1	τ(10)=-0.05, p=0.86
IP-10	1	1	τ(7)=-0.053, p=0.874
Eotaxin	1	1	τ(7)=-0.053, p=0.874
IL-1a	0	5	τ(9)=-0.07, p=0.82
IL-10	1	1	τ(7)=-0.073, p=0.842
IL-6	1	2	τ(7)=-0.098, p=0.761
IL-5	0	5	τ(9)=-0.1, p=0.73
IL-1a	0	4	τ(9)=-0.12, p=0.67
G-CSF	0	3	τ(9)=-0.13, p=0.65
IL-15	1	1	τ(7)=-0.159, p=0.634
KC	0	2	τ(9)=-0.18, p=0.51
IL-6	0	2	τ(9)=-0.18, p=0.51
IP-10	1	3	τ(4)=-0.183, p=0.718
IL-5	1	4	τ(4)=-0.183, p=0.718
RANTES	1	3	τ(4)=-0.183, p=0.718
IL-15	1	3	τ(4)=-0.183, p=0.718

IL-1a	1	4	τ(4)=-0.183, p=0.718
IL-17	1	4	τ(4)=-0.183, p=0.718
Eotaxin	1	3	τ(4)=-0.183, p=0.718
Eotaxin	1	4	τ(4)=-0.183, p=0.718
IP-10	1	2	τ(7)=-0.195, p=0.543
IL-5	1	5	τ(4)=-0.236, p=0.655
IL-1a	1	5	τ(4)=-0.236, p=0.655
IL-17	1	5	τ(4)=-0.236, p=0.655
RANTES	0	3	τ(9)=-0.26, p=0.37
KC	0	3	τ(9)=-0.26, p=0.37
G-CSF	0	5	τ(9)=-0.26, p=0.37
IL-2	0	2	τ(9)=-0.26, p=0.423
RANTES	1	2	τ(6)=-0.276, p=0.444
IL-10	0	1	τ(10)=-0.28, p=0.35
Eotaxin	1	2	τ(7)=-0.293, p=0.362
IL-1a	0	2	τ(9)=-0.3, p=0.28
IFNy	0	3	τ(9)=-0.35, p=0.3
G-CSF	0	4	τ(9)=-0.37, p=0.19
IFNy	0	1	τ(10)=-0.38, p=0.21
IL-2	0	3	τ(9)=-0.42, p=0.21
IL-2	0	4	τ(9)=-0.45, p=0.16
IL-2	0	5	τ(9)=-0.49, p=0.15
RANTES	1	1	τ(6)=-0.545, p=0.15

IP-10	1	4	τ(4)=-0.548, p=0.279
RANTES	1	4	τ(4)=-0.548, p=0.279
IL-15	1	4	τ(4)=-0.548, p=0.279
TNFa	1	1	τ(7)=-0.594, p=0.116
IP-10	1	5	τ(4)=-0.707, p=0.18
RANTES	1	5	τ(4)=-0.707, p=0.18
IL-15	1	5	τ(4)=-0.707, p=0.18
Eotaxin	1	5	τ(4)=-0.707, p=0.18
G-CSF	0	1	τ(10)=-0.44, p=0.1
IL-1b	0	1	τ(10)=-0.6, p=0.02

Table S9. Cytokine levels correlate with hormone levels

Cytokine	Day of Bleed	Hormone	Kendall's Tau Analysis
IL-1b	1	PRL	τ(7)=0.81, p=0.01
IFNy	1	LH	τ(7)=0.59, p=0.09
IL-10	0	FSH	τ(10)=0.585, p=0.038
IL-1b	0	FSH	τ(10)=0.489, p=0.056
IP-10	1	Progesterone	τ(4)=0.67, p=0.17
IL-5	1	Progesterone	τ(4)=0.67, p=0.17
KC	1	Progesterone	τ(4)=0.67, p=0.17
IL-1a	1	Progesterone	τ(4)=0.67, p=0.17
IL-17	1	Progesterone	τ(4)=0.67, p=0.17
IFNy	1	PRL	τ(7)=0.46, p=0.18
IL-4	0	FSH	τ(10)=0.45, p=0.12
TNFa	0	FSH	τ(10)=0.45, p=0.12
IL-5	0	FSH	τ(10)=0.39, p=0.13
GM-CSF	1	PRL	τ(7)=0.36, p=0.32
GM-CSF	1	LH	τ(7)=0.36, p=0.32
TNFa	1	FSH	τ(7)=0.36, p=0.32
IL-2	1	LH	τ(7)=0.36, p=0.32
IL-2	1	PRL	τ(7)=0.36, p=0.32
IL-10	0	LH	τ(10)=0.33, p=0.24
KC	1	PRL	τ(7)=0.33, p=0.29
IL-6	1	LH	τ(7)=0.33, p=0.29

G-CSF	1	PRL	τ(7)=0.33, p=0.29
G-CSF	1	LH	τ(7)=0.33, p=0.29
Eotaxin	1	LH	τ(7)=0.33, p=0.29
Eotaxin	1	FSH	τ(7)=0.33, p=0.29
RANTES	1	FSH	τ(6)=0.33, p=0.35
IL-6	1	Progesterone	τ(4)=0.33, p=0.5
IL-1b	0	PRL	τ(10)=0.32, p=0.2
IL-5	0	PRL	τ(10)=0.32, p=0.21
G-CSF	0	LH	τ(10)=0.29, p=0.25
IL-1b	0	LH	τ(10)=0.28, p=0.28
IL-15	0	PRL	τ(10)=0.24, p=0.36
IP-10	1	LH	τ(7)=0.24, p=0.45
IL-1b	1	LH	τ(7)=0.24, p=0.45
IL-1a	1	PRL	τ(7)=0.24, p=0.45
IL-5	1	LH	τ(7)=0.24, p=0.45
IL-17	1	PRL	τ(7)=0.24, p=0.45
IL-6	0	FSH	τ(9)=0.2, p=0.46
IL-10	1	FSH	τ(7)=0.2, p=0.57
IL-1a	0	PRL	τ(10)=0.16, p=0.53
IL-4	0	PRL	τ(10)=0.15, p=0.6
TNFa	0	PRL	τ(10)=0.15, p=0.6
IL-17	0	PRL	τ(10)=0.14, p=0.59
IL-15	1	FSH	τ(7)=0.14, p=0.65

IL-6	0	LH	$\tau(9)=0.11$, $p=0.68$
IL-10	0	PRL	$\tau(10)=0.11$, $p=0.7$
IL-10	1	PRL	$\tau(7)=0.07$, $p=0.85$
IL-15	0	FSH	$\tau(10)=0.05$, $p=0.86$
IL-4	0	LH	$\tau(10)=0.05$, $p=0.86$
IL-1a	0	FSH	$\tau(10)=0.05$, $p=0.86$
TNFa	0	LH	$\tau(10)=0.05$, $p=0.86$
G-CSF	0	FSH	$\tau(10)=0.05$, $p=0.86$
IL-1a	1	LH	$\tau(7)=0.05$, $p=0.88$
IP-10	1	FSH	$\tau(7)=0.05$, $p=0.88$
IL-1a	0	LH	$\tau(10)=-0.02$, $p=0.93$
IL-5	0	LH	$\tau(10)=-0.05$, $p=0.86$
IL-2	0	LH	$\tau(10)=-0.05$, $p=0.86$
IFNy	0	LH	$\tau(10)=-0.05$, $p=0.86$
KC	1	LH	$\tau(7)=-0.05$, $p=0.88$
IL-6	1	PRL	$\tau(7)=-0.05$, $p=0.88$
RANTES	0	LH	$\tau(10)=-0.07$, $p=0.79$
KC	0	LH	$\tau(10)=-0.07$, $p=0.79$
IL-10	1	LH	$\tau(7)=-0.07$, $p=0.85$
IL-17	1	LH	$\tau(7)=-0.14$, $p=0.65$
IP-10	1	PRL	$\tau(7)=-0.14$, $p=0.65$
IL-5	1	FSH	$\tau(7)=-0.14$, $p=0.65$
IL-5	1	PRL	$\tau(7)=-0.14$, $p=0.65$

IL-2	0	PRL	τ(10)=-0.15, p=0.6
RANTES	0	PRL	τ(10)=-0.16, p=0.53
G-CSF	0	PRL	τ(10)=-0.16, p=0.53
KC	0	FSH	τ(10)=-0.18, p=0.47
IFNy	1	FSH	τ(7)=-0.2, p=0.57
IL-15	1	LH	τ(7)=-0.24, p=0.45
Eotaxin	1	PRL	τ(7)=-0.24, p=0.45
IL-17	0	FSH	τ(10)=-0.25, p=0.32
IP-10	0	FSH	τ(10)=-0.27, p=0.28
RANTES	0	FSH	τ(10)=-0.27, p=0.28
Eotaxin	0	FSH	τ(10)=-0.27, p=0.28
IL-15	0	LH	τ(10)=-0.29, p=0.25
Eotaxin	0	PRL	τ(10)=-0.29, p=0.25
KC	0	PRL	τ(10)=-0.33, p=0.18
IL-1a	1	FSH	τ(7)=-0.33, p=0.29
G-CSF	1	Progesterone	τ(4)=-0.33, p=0.5
IL-17	0	LH	τ(10)=-0.36, p=0.15
TNFa	1	PRL	τ(7)=-0.36, p=0.32
IFNy	0	FSH	τ(10)=-0.4, p=0.16
IL-15	1	PRL	τ(7)=-0.43, p=0.18
IL-6	1	FSH	τ(7)=-0.43, p=0.18
G-CSF	1	FSH	τ(7)=-0.43, p=0.18
IL-2	0	FSH	τ(10)=-0.45, p=0.12

IFNy	0	PRL	τ(10)=-0.45, p=0.12
RANTES	1	PRL	τ(6)=-0.47, p=0.19
RANTES	1	LH	τ(6)=-0.47, p=0.19
TNFa	1	LH	τ(7)=-0.54, p=0.13
IL-10	1	Progesterone	τ(4)=-0.71, p=0.18
IL-2	1	Progesterone	τ(4)=-0.71, p=0.18
IFNy	1	Progesterone	τ(4)=-0.71, p=0.18
GM-CSF	1	Progesterone	τ(4)=-0.71, p=0.18
IP-10	0	PRL	τ(10)=-0.467, p=0.06
IL-1b	1	FSH	τ(7)=-0.524, p=0.099
IL-17	1	FSH	τ(7)=-0.52, p=0.1
Eotaxin	0	LH	τ(10)=-0.556, p=0.025
KC	1	FSH	τ(7)=-0.62, p=0.05
IP-10	0	LH	τ(10)=-0.733, p=0.003

Table S10. Cytokine levels correlate with immunoglobulin levels			
**Cytokine**	**Day of Bleed**	**Ig subtype**	**Kendall's Tau Analysis**
IL-6	1	IgA	τ(7)=0.78, p=0.015
G-CSF	1	IgA	τ(7)=0.78, p=0.015
KC	1	IgA	τ(7)=0.68, p=0.033
IL-15	1	IgG2a	τ(7)=0.62, p=0.065
IL-17	1	IgG2a	τ(7)=0.62, p=0.065
IL-5	1	IgA	τ(7)=0.59, p=0.068
IL-1a	0	IgG1	τ(10)=0.56, p=0.03
IL-1a	0	IgG1	τ(10)=0.56, p=0.025
KC	1	IgG3	τ(7)=0.52, p=0.099
KC	1	IgM	τ(7)=0.52, p=0.099
IL-6	1	IgG3	τ(7)=0.52, p=0.099
G-CSF	1	IgG3	τ(7)=0.52, p=0.099
IL-5	1	IgG2a	τ(7)=0.51, p=0.132
KC	1	IgG2a	τ(7)=0.51, p=0.132
IL-4	0	IgG2a	τ(10)=0.5, p=0.101
TNFa	0	IgG2a	τ(10)=0.5, p=0.101
IFNy	0	IgA	τ(10)=0.49, p=0.105
IFNy	1	IgA	τ(7)=0.47, p=0.179
IL-4	0	IgM	τ(10)=0.45, p=0.117
IL-4	0	IgG3	τ(10)=0.45, p=0.117
IL-2	0	IgG1	τ(10)=0.45, p=0.117

TNFa	0	IgG3	τ(10)=0.45, p=0.117
TNFa	0	IgM	τ(10)=0.45, p=0.117
IL-5	1	IgG3	τ(7)=0.43, p=0.176
IL-6	1	IgG2b	τ(7)=0.43, p=0.176
Eotaxin	1	IgG2b	τ(7)=0.43, p=0.176
IL-17	0	IgG2a	τ(10)=0.43, p=0.108
IL-1b	0	IgA	τ(10)=0.43, p=0.108
IL-5	0	IgM	τ(10)=0.41, p=0.106
IL-1a	1	IgG2a	τ(7)=0.39, p=0.241
IL-17	1	IgA	τ(7)=0.39, p=0.224
IFNy	0	IgG2a	τ(10)=0.39, p=0.203
IL-4	0	IgA	τ(10)=0.38, p=0.207
TNFa	0	IgA	τ(10)=0.38, p=0.207
IL-5	0	IgG3	τ(10)=0.36, p=0.151
IL-17	0	IgM	τ(10)=0.36, p=0.151
IP-10	0	IgG2a	τ(10)=0.35, p=0.187
Eotaxin	0	IgG2a	τ(10)=0.348, p=0.187
IL-1a	0	IgG2b	τ(10)=0.33, p=0.18
IL-5	1	IgG2b	τ(7)=0.333, p=0.293
IL-6	1	IgM	τ(7)=0.333, p=0.293
G-CSF	1	IgM	τ(7)=0.333, p=0.293
RANTES	1	IgG1	τ(6)=0.333, p=0.348
RANTES	0	IgG2a	τ(10)=0.298, p=0.258

IP-10	0	IgM	τ(10)=0.289, p=0.245
Eotaxin	0	IgM	τ(10)=0.289, p=0.245
IL-15	0	IgM	τ(10)=0.29, p=0.245
IL-10	0	IgG2a	τ(10)=0.283, p=0.343
IP-10	1	IgG2a	τ(7)=0.282, p=0.402
RANTES	1	IgG2a	τ(6)=0.258, p=0.499
IL-1b	0	IgG2a	τ(10)=0.257, p=0.34
IL-15	0	IgG2a	τ(10)=0.25, p=0.346
IL-5	1	IgM	τ(7)=0.238, p=0.453
IL-15	1	IgG2b	τ(7)=0.238, p=0.453
IL-17	1	IgG3	τ(7)=0.238, p=0.453
IL-17	1	IgM	τ(7)=0.238, p=0.453
IL-10	0	IgA	τ(10)=0.236, p=0.421
IFNy	1	IgG3	τ(7)=0.197, p=0.568
IL-1a	1	IgA	τ(7)=0.195, p=0.543
IL-1b	1	IgA	τ(7)=0.195, p=0.543
IP-10	0	IgA	τ(10)=0.193, p=0.456
IL-2	1	IgA	τ(7)=0.183, p=0.614
GM-CSF	1	IgA	τ(7)=0.183, p=0.614
IL-10	0	IgM	τ(10)=0.181, p=0.517
IL-2	1	IgG3	τ(7)=0.178, p=0.617
TNFa	1	IgG1	τ(7)=0.178, p=0.617
GM-CSF	1	IgG3	τ(7)=0.178, p=0.617

IL-6	1	IgG2a	τ(7)=0.169, p=0.615
IP-10	0	IgG3	τ(10)=0.156, p=0.531
RANTES	0	IgM	τ(10)=0.156, p=0.531
IL-15	0	IgG3	τ(10)=0.16, p=0.531
IP-10	1	IgG2b	τ(7)=0.143, p=0.652
IL-15	1	IgM	τ(7)=0.143, p=0.652
IL-1b	0	IgM	τ(10)=0.138, p=0.586
IL-17	0	IgG3	τ(10)=0.135, p=0.59
IL-1a	0	IgM	τ(10)=0.11, p=0.655
RANTES	0	IgG3	τ(10)=0.111, p=0.655
IL-6	0	IgG3	τ(10)=0.111, p=0.655
IL-10	0	IgG3	τ(10)=0.108, p=0.698
Eotaxin	1	IgA	τ(7)=0.098, p=0.761
IL-1b	0	IgG3	τ(10)=0.092, p=0.717
IL-5	0	IgG2b	τ(10)=0.09, p=0.719
IL-5	0	IgG2a	τ(10)=0.075, p=0.777
IL-6	0	IgM	τ(10)=0.067, p=0.788
RANTES	1	IgG2b	τ(6)=0.067, p=0.851
IL-1a	0	IgG3	τ(10)=0.07, p=0.788
IL-10	1	IgG3	τ(7)=0.066, p=0.849
G-CSF	1	IgG2a	τ(7)=0.056, p=0.867
Eotaxin	1	IgG2a	τ(7)=0.056, p=0.867
IL-2	0	IgG2b	τ(10)=0.05, p=0.862

RANTES	0	IgA	τ(10)=0.048, p=0.852
G-CSF	0	IgA	τ(10)=0.048, p=0.852
KC	1	IgG2b	τ(7)=0.048, p=0.881
IL-1a	1	IgM	τ(7)=0.048, p=0.881
IL-1b	1	IgG3	τ(7)=0.048, p=0.881
IL-1b	1	IgM	τ(7)=0.048, p=0.881
IL-6	1	IgG1	τ(7)=0.048, p=0.881
G-CSF	1	IgG2b	τ(7)=0.048, p=0.881
IgG2b	0	IL-1b	τ(10)=0.046, p=0.856
IL-17	0	IgA	τ(10)=0.024, p=0.926
IP-10	0	IgG2b	τ(10)=-0.022, p=0.929
Eotaxin	0	IgG3	τ(10)=-0.022, p=0.929
IL-5	0	IgA	τ(10)=-0.024, p=0.926
IL-5	0	IgG1	τ(10)=-0.045, p=0.857
IP-10	1	IgG1	τ(7)=-0.048, p=0.881
IL-15	1	IgG3	τ(7)=-0.048, p=0.881
IL-1a	1	IgG1	τ(7)=-0.048, p=0.881
IL-1b	1	IgG1	τ(7)=-0.048, p=0.881
IL-17	1	IgG2b	τ(7)=-0.048, p=0.881
Eotaxin	1	IgG3	τ(7)=-0.048, p=0.881
IL-4	0	IgG2b	τ(10)=-0.05, p=0.862
TNFa	0	IgG2b	τ(10)=-0.05, p=0.862
IFNy	1	IgG2b	τ(7)=-0.066, p=0.849

IFNy	1	IgM	τ(7)=-0.066, p=0.849
IP-10	0	IgG1	τ(10)=-0.067, p=0.788
IL-6	0	IgG2b	τ(10)=-0.067, p=0.788
IFNy	1	IgG2a	τ(7)=-0.078, p=0.833
IL-1b	0	IgG1	τ(10)=-0.092, p=0.717
IL-15	1	IgA	τ(7)=-0.098, p=0.761
IL-6	0	IgG2a	τ(10)=-0.099, p=0.706
G-CSF	0	IgG2a	τ(10)=-0.099, p=0.706
KC	0	IgG1	τ(10)=-0.111, p=0.655
IL-6	0	IgG1	τ(10)=-0.111, p=0.655
G-CSF	0	IgG1	τ(10)=-0.111, p=0.655
IP-10	1	IgG3	τ(7)=-0.143, p=0.652
IP-10	1	IgM	τ(7)=-0.143, p=0.652
IL-15	1	IgG1	τ(7)=-0.143, p=0.652
IL-1a	1	IgG3	τ(7)=-0.143, p=0.652
Eotaxin	1	IgG1	τ(7)=-0.143, p=0.652
IFNy	0	IgM	τ(10)=-0.149, p=0.602
RANTES	0	IgG2b	τ(10)=-0.156, p=0.531
KC	0	IgG3	τ(10)=-0.156, p=0.531
IL-1b	1	IgG2a	τ(7)=-0.169, p=0.615
IL-2	1	IgG2b	τ(7)=-0.178, p=0.617
IL-2	1	IgM	τ(7)=-0.178, p=0.617
TNFa	1	IgG3	τ(7)=-0.178, p=0.617

GM-CSF	1	IgG2b	τ(7)=-0.178, p=0.617
GM-CSF	1	IgM	τ(7)=-0.178, p=0.617
IL-17	0	IgG2b	τ(10)=-0.18, p=0.472
IL-10	0	IgG2b	τ(10)=-0.181, p=0.517
IL-10	1	IgG2b	τ(7)=-0.197, p=0.568
IL-10	1	IgM	τ(7)=-0.197, p=0.568
KC	0	IgM	τ(10)=-0.2, p=0.421
IL-15	0	IgG2b	τ(10)=-0.2, p=0.421
IL-10	1	IgA	τ(7)=-0.202, p=0.565
TNFa	1	IgG2a	τ(7)=-0.211, p=0.581
IL-5	1	IgG1	τ(7)=-0.238, p=0.453
IL-1a	1	IgG2b	τ(7)=-0.238, p=0.453
IL-17	1	IgG1	τ(7)=-0.238, p=0.453
Eotaxin	1	IgM	τ(7)=-0.238, p=0.453
IL-6	0	IgA	τ(10)=-0.242, p=0.352
KC	0	IgG2b	τ(10)=-0.244, p=0.325
G-CSF	0	IgG2b	τ(10)=-0.244, p=0.325
Eotaxin	0	IgG1	τ(10)=-0.244, p=0.325
IL-15	0	IgG1	τ(10)=-0.24, p=0.325
IFNy	0	IgG2b	τ(10)=-0.248, p=0.384
Eotaxin	0	IgG2b	τ(10)=-0.289, p=0.245
IL-15	0	IgA	τ(10)=-0.29, p=0.264
IL-1a	0	IgA	τ(10)=-0.29, p=0.264

IL-10	1	IgG1	τ(7)=-0.329, p=0.342
KC	1	IgG1	τ(7)=-0.333, p=0.293
G-CSF	1	IgG1	τ(7)=-0.333, p=0.293
IL-1a	0	IgG2a	τ(10)=-0.348, p=0.187
IFNy	0	IgG1	τ(10)=-0.348, p=0.223
IFNy	0	IgG3	τ(10)=-0.348, p=0.223
IL-17	0	IgG1	τ(10)=-0.36, p=0.151
IL-2	0	IgA	τ(10)=-0.379, p=0.207
KC	0	IgG2a	τ(10)=-0.398, p=0.132
IL-10	0	IgG1	τ(10)=-0.398, p=0.154
IL-2	1	IgG2a	τ(7)=-0.422, p=0.27
GM-CSF	1	IgG2a	τ(7)=-0.422, p=0.27
IL-4	0	IgG1	τ(10)=-0.447, p=0.117
IL-2	0	IgG3	τ(10)=-0.447, p=0.117
IL-2	0	IgM	τ(10)=-0.447, p=0.117
TNFa	0	IgG1	τ(10)=-0.447, p=0.117
IFNy	1	IgG1	τ(7)=-0.461, p=0.183
RANTES	1	IgM	τ(10)=-0.467, p=0.06
IL-2	0	IgG2a	τ(10)=-0.467, p=0.06
IL-2	1	IgG1	τ(10)=-0.467, p=0.06
GM-CSF	1	IgG1	τ(6)=-0.467, p=0.188
IL-10	1	IgG2a	τ(10)=-0.5, p=0.101
TNFa	1	IgA	τ(10)=-0.511, p=0.04

RANTES	1	IgA	τ(10)=-0.511, p=0.04
RANTES	0	IgG1	τ(7)=-0.535, p=0.134
G-CSF	0	IgM	τ(7)=-0.535, p=0.134
G-CSF	0	IgM	τ(7)=-0.545, p=0.141
G-CSF	0	IgG3	τ(7)=-0.548, p=0.13
G-CSF	0	IgG3	τ(6)=-0.552, p=0.126
IL-1b	1	IgG2b	τ(7)=-0.619, p=0.051
RANTES	1	IgG3	τ(6)=-0.733, p=0.039

Table S11. Cytokine levels correlate with cytokine Levels			
**Cytokine**	**Day of Bleed**	**Cytokine**	**Kendall's Tau Analysis**
IL-4	0	TNF-a	τ(10)=1, p=0.003
IL-2	1	GM-CSF	τ(7)=1, p=0.014
IL-2	1	IFNy	τ(7)=0.739, p=0.061
IL-10	1	GM-CSF	τ(7)=0.739, p=0.061
IFNy	1	GM-CSF	τ(7)=0.739, p=0.061
IL-10	1	IL-2	τ(7)=0.739, p=0.061
IL-10	0	IL-4	τ(10)=0.728, p=0.025
TNF-a	0	IL-10	τ(10)=0.728, p=0.025
G-CSF	1	IFNy	τ(7)=0.724, p=0.037
KC	1	IL-17	τ(7)=0.714, p=0.024
IL-1b	0	IL-10	τ(10)=0.636, p=0.026
IL-5	1	IL-17	τ(7)=0.619, p=0.051
IL-1a	1	IL-17	τ(7)=0.619, p=0.051
IL-6	1	G-CSF	τ(7)=0.619, p=0.051
RANTES	1	IL-15	τ(6)=0.6, p=0.091
Eotaxin	0	IP-10	τ(10)=0.556, p=0.025
RANTES	0	IL-17	τ(10)=0.539, p=0.031
IL-5	1	KC	τ(7)=0.535, p=0.134
IP-10	1	Eotaxin	τ(7)=0.535, p=0.134
IL-5	1	Eotaxin	τ(7)=0.524, p=0.099
IL-5	1	IL-6	τ(7)=0.524, p=0.099
IL-6	0	RANTES	τ(7)=0.524, p=0.099

KC	0	IL-6	τ(7)=0.524, p=0.099
IL-5	0	IL-15	τ(7)=0.524, p=0.099
IL-15	0	IL-17	τ(7)=0.49236596, p=0.212
IL-15	1	IP-10	τ(10)=0.467, p=0.06
G-CSF	1	IL-2	τ(10)=0.467, p=0.06
GM-CSF	1	G-CSF	τ(10)=0.463, p=0.113
TNFa	1	IL-10	τ(10)=0.463, p=0.113
IL-1b	0	IL-4	τ(7)=0.461, p=0.183
TNFa	0	IL-1b	τ(10)=0.452, p=0.116
IFNy	1	IL-6	τ(10)=0.452, p=0.116
IL-17	0	IL-4	τ(10)=0.452, p=0.116
TNFa	0	IL-5	τ(10)=0.45, p=0.12
TNFa	0	IL-17	τ(10)=0.45, p=0.12
IL-5	0	IL-4	τ(10)=0.449, p=0.072
RANTES	0	IL-4	τ(10)=0.449, p=0.072
TNFa	0	RANTES	τ(10)=0.447, p=0.117
IFNy	0	IP-10	τ(10)=0.447, p=0.117
IL-5	1	IP-10	τ(7)=0.429, p=0.176
IL-1a	1	IP-10	τ(7)=0.429, p=0.176
IL-1b	1	IL-1a	τ(7)=0.429, p=0.176
IL-17	1	IP-10	τ(7)=0.429, p=0.176
IL-6	1	KC	τ(7)=0.429, p=0.176
G-CSF	1	KC	τ(7)=0.429, p=0.176

IL-17	0	IL-5	τ(10)=0.386, p=0.125
Eotaxin	0	IL-15	τ(10)=0.378, p=0.128
IL-15	0	IL-4	τ(10)=0.35, p=0.22
IL-1a	0	IL-2	τ(10)=0.35, p=0.22
IL-6	0	IL-4	τ(10)=0.348, p=0.223
TNFa	0	IL-15	τ(10)=0.348, p=0.223
TNFa	0	IL-6	τ(10)=0.348, p=0.223
G-CSF	0	IL-2	τ(10)=0.348, p=0.223
IFNy	0	G-CSF	τ(10)=0.348, p=0.223
TNFa	1	RANTES	τ(6)=0.346, p=0.38
KC	1	IP-10	τ(7)=0.333, p=0.293
IL-15	1	IL-5	τ(7)=0.333, p=0.293
IL-1a	1	KC	τ(7)=0.333, p=0.293
IL-1a	1	IL-15	τ(7)=0.333, p=0.293
IL-1b	1	KC	τ(7)=0.333, p=0.293
IL-17	1	IL-15	τ(7)=0.333, p=0.293
G-CSF	1	IL-5	τ(7)=0.333, p=0.293
RANTES	1	IP-10	τ(6)=0.333, p=0.348
IL-1a	1	RANTES	τ(6)=0.333, p=0.348
IFNy	1	IL-5	τ(7)=0.329, p=0.342
IFNy	1	KC	τ(7)=0.329, p=0.342
IFNy	1	Eotaxin	τ(7)=0.329, p=0.342
KC	0	RANTES	τ(10)=0.29, p=0.25

Eotaxin	0	IL-17	τ(10)=0.27, p=0.281
IL-6	0	IL-10	τ(10)=0.253, p=0.365
IFNy	0	RANTES	τ(10)=0.248, p=0.384
IFNy	0	Eotaxin	τ(10)=0.248, p=0.384
IL-15	1	KC	τ(7)=0.238, p=0.453
IL-1a	1	IL-5	τ(7)=0.238, p=0.453
IL-17	1	IL-1b	τ(7)=0.238, p=0.453
Eotaxin	1	IL-6	τ(7)=0.238, p=0.453
IL-1a	0	IL-5	τ(10)=0.23, p=0.37
IL-6	0	IL-17	τ(10)=0.225, p=0.369
IL-17	1	RANTES	τ(6)=0.2, p=0.573
IFNy	1	IL-1b	τ(7)=0.197, p=0.568
IFNy	1	IL-17	τ(7)=0.197, p=0.568
Eotaxin	0	IL-10	τ(10)=0.181, p=0.517
IL-15	0	IL-10	τ(10)=0.18, p=0.52
IL-6	1	IL-2	τ(7)=0.178, p=0.617
Eotaxin	1	IL-2	τ(7)=0.178, p=0.617
GM-CSF	1	IL-6	τ(7)=0.178, p=0.617
GM-CSF	1	Eotaxin	τ(7)=0.178, p=0.617
RANTES	0	IP-10	τ(10)=0.16, p=0.53
IL-15	0	RANTES	τ(10)=0.16, p=0.53
KC	0	IL-2	τ(10)=0.15, p=0.6
IL-6	1	IP-10	τ(7)=0.143, p=0.652

IL-6	1	IL-17	τ(7)=0.143, p=0.652
G-CSF	1	IL-1b	τ(7)=0.143, p=0.652
G-CSF	1	IL-17	τ(7)=0.143, p=0.652
Eotaxin	1	IL-17	τ(7)=0.143, p=0.652
IL-1b	0	IL-5	τ(10)=0.116, p=0.649
IFNy	0	IL-2	τ(10)=0.111, p=0.739
IP-10	0	IL-10	τ(10)=0.11, p=0.7
IL-5	0	IL-10	τ(10)=0.11, p=0.7
G-CSF	0	IL-1b	τ(10)=0.092, p=0.717
IFNy	1	IL-10	τ(7)=0.091, p=0.812
KC	0	IP-10	τ(10)=0.07, p=0.79
IL-15	0	KC	τ(10)=0.07, p=0.79
IL-6	0	IL-15	τ(10)=0.067, p=0.788
G-CSF	0	IP-10	τ(10)=0.067, p=0.788
Eotaxin	0	KC	τ(10)=0.067, p=0.788
Eotaxin	1	RANTES	τ(6)=0.067, p=0.851
G-CSF	1	IL-10	τ(7)=0.066, p=0.849
IFNy	1	IP-10	τ(7)=0.066, p=0.849
IP-10	0	IL-4	τ(10)=0.05, p=0.86
RANTES	0	IL-5	τ(10)=0.05, p=0.86
TNFa	0	IP-10	τ(10)=0.05, p=0.862
Eotaxin	0	IL-4	τ(10)=0.05, p=0.862
Eotaxin	0	TNFa	τ(10)=0.05, p=0.862

IL-1b	1	IP-10	τ(7)=0.048, p=0.881
Eotaxin	1	KC	τ(7)=0.048, p=0.881
Eotaxin	1	IL-15	τ(7)=0.048, p=0.881
Eotaxin	1	G-CSF	τ(7)=0.048, p=0.881
IL-17	0	IP-10	τ(10)=0.045, p=0.857
IL-17	0	KC	τ(10)=0.045, p=0.857
RANTES	0	IL-10	τ(10)=0.04, p=0.9
IL-17	0	IL-10	τ(10)=0.037, p=0.897
G-CSF	0	IL-10	τ(10)=0.036, p=0.897
Eotaxin	0	IL-1a	τ(10)=0.022, p=0.929
IL-15	0	IP-10	τ(10)=0.02, p=0.93
IL-1a	0	IL-15	τ(10)=0.02, p=0.93
IL-1a	0	KC	τ(10)=-0.02, p=0.93
Eotaxin	0	RANTES	τ(10)=-0.022, p=0.929
Eotaxin	0	G-CSF	τ(10)=-0.022, p=0.929
IL-6	0	IL-5	τ(10)=-0.045, p=0.857
Eotaxin	0	IL-5	τ(10)=-0.045, p=0.857
IL-1b	0	IP-10	τ(10)=-0.046, p=0.856
IL-1b	0	IL-15	τ(10)=-0.046, p=0.856
IL-6	1	IL-1a	τ(7)=-0.048, p=0.881
IL-6	1	IL-1b	τ(7)=-0.048, p=0.881
G-CSF	1	IL-1a	τ(7)=-0.048, p=0.881
Eotaxin	1	IL-1a	τ(7)=-0.048, p=0.881

IP-10	0	IL-2	τ(10)=-0.05, p=0.86
KC	0	IL-5	τ(10)=-0.05, p=0.86
Eotaxin	0	IL-2	τ(10)=-0.05, p=0.862
IFNy	0	KC	τ(10)=-0.05, p=0.862
IFNy	0	IL-1b	τ(10)=-0.051, p=0.86
Eotaxin	1	IL-10	τ(7)=-0.066, p=0.849
IFNy	1	IL-1a	τ(7)=-0.066, p=0.849
RANTES	1	IL-5	τ(6)=-0.067, p=0.851
IL-17	0	IL-1b	τ(10)=-0.07, p=0.785
IL-17	0	IL-1a	τ(10)=-0.09, p=0.719
IL-1b	0	IL-1a	τ(10)=-0.092, p=0.717
Eotaxin	0	IL-1b	τ(10)=-0.092, p=0.717
IL-6	0	IP-10	τ(10)=-0.111, p=0.655
IL-6	0	IL-1a	τ(10)=-0.111, p=0.655
Eotaxin	0	IL-6	τ(10)=-0.111, p=0.655
IFNy	0	IL-4	τ(10)=-0.111, p=0.739
IFNy	0	TNFa	τ(10)=-0.111, p=0.739
IL-6	0	IL-1b	τ(10)=-0.138, p=0.586
IL-1b	1	IL-5	τ(7)=-0.143, p=0.652
IL-6	1	IL-15	τ(7)=-0.143, p=0.652
TNFa	0	KC	τ(10)=-0.149, p=0.602
KC	0	IL-4	τ(10)=-0.15, p=0.6
G-CSF	0	KC	τ(10)=-0.156, p=0.531

IL-1a	0	IP-10	τ(10)=-0.16, p=0.53
TNFa	1	IL-2	τ(7)=-0.1666667, p=0.683
GM-CSF	1	TNFa	τ(7)=-0.167, p=0.683
IL-17	1	IL-2	τ(7)=-0.178, p=0.617
GM-CSF	1	IL-17	τ(7)=-0.178, p=0.617
KC	0	IL-10	τ(10)=-0.18, p=0.52
IL-1b	1	IL-10	τ(7)=-0.197, p=0.568
IL-6	1	IL-10	τ(7)=-0.197, p=0.568
KC	1	RANTES	τ(6)=-0.2, p=0.573
IL-5	0	IP-10	τ(10)=-0.23, p=0.37
IL-1b	1	IL-15	τ(7)=-0.238, p=0.453
G-CSF	1	IP-10	τ(7)=-0.238, p=0.453
Eotaxin	1	IL-1b	τ(7)=-0.238, p=0.453
G-CSF	0	RANTES	τ(10)=-0.244, p=0.325
IFNy	0	IL-6	τ(10)=-0.248, p=0.384
IL-1a	0	IL-10	τ(10)=-0.25, p=0.37
RANTES	1	IL-10	τ(6)=-0.258, p=0.499
IL-1b	0	RANTES	τ(10)=-0.276, p=0.276
G-CSF	0	IL-15	τ(10)=-0.289, p=0.245
G-CSF	0	IL-1a	τ(10)=-0.289, p=0.245
IL-5	1	IL-10	τ(7)=-0.329, p=0.342
KC	1	IL-10	τ(7)=-0.329, p=0.342
IFNy	1	IL-15	τ(7)=-0.329, p=0.342

G-CSF	0	IL-6	τ(10)=-0.333, p=0.18
G-CSF	1	IL-15	τ(7)=-0.333, p=0.293
IL-1b	1	RANTES	τ(6)=-0.333, p=0.348
IL-6	0	IL-2	τ(10)=-0.348, p=0.223
TNFa	0	IL-1a	τ(10)=-0.348, p=0.223
G-CSF	0	IL-4	τ(10)=-0.348, p=0.223
G-CSF	0	TNFa	τ(10)=-0.348, p=0.223
IFNy	0	IL-15	τ(10)=-0.348, p=0.223
IL-15	0	IL-2	τ(10)=-0.35, p=0.22
IL-1a	0	IL-4	τ(10)=-0.35, p=0.22
IP-10	1	IL-2	τ(7)=-0.356, p=0.317
TNFa	1	IL-1a	τ(7)=-0.356, p=0.317
TNFa	1	IL-1b	τ(7)=-0.356, p=0.317
Eotaxin	1	TNFa	τ(7)=-0.356, p=0.317
GM-CSF	1	IP-10	τ(7)=-0.356, p=0.317
G-CSF	0	IL-5	τ(10)=-0.405, p=0.106
IFNy	0	IL-1a	τ(10)=-0.447, p=0.117
IL-5	0	IL-2	τ(10)=-0.45, p=0.12
RANTES	0	IL-2	τ(10)=-0.45, p=0.12
IL-17	0	IL-2	τ(10)=-0.452, p=0.116
IFNy	0	IL-5	τ(10)=-0.452, p=0.116
IL-15	1	IL-10	τ(7)=-0.461, p=0.183
IL-17	1	IL-10	τ(7)=-0.461, p=0.183

IL-1b	0	IL-2	τ(10)=-0.463, p=0.113
IL-6	1	RANTES	τ(10)=-0.467, p=0.06
IL-15	1	IL-2	τ(6)=-0.467, p=0.188
IL-1a	1	IL-2	τ(7)=-0.535, p=0.134
TNFa	1	IP-10	τ(7)=-0.535, p=0.134
TNFa	1	IL-5	τ(7)=-0.535, p=0.134
TNFa	1	KC	τ(7)=-0.535, p=0.134
TNFa	1	IL-17	τ(7)=-0.535, p=0.134
TNFa	1	IL-6	τ(7)=-0.535, p=0.134
G-CSF	1	TNFa	τ(7)=-0.535, p=0.134
GM-CSF	1	IL-15	τ(7)=-0.535, p=0.134
GM-CSF	1	IL-1a	τ(7)=-0.535, p=0.134
RANTES	1	IL-2	τ(7)=-0.535, p=0.134
GM-CSF	1	RANTES	τ(10)=-0.539, p=0.031
IFNy	1	RANTES	τ(6)=-0.577, p=0.143
RANTES	0	IL-1a	τ(6)=-0.577, p=0.143
G-CSF	0	IL-17	τ(7)=-0.592, p=0.087
IL-10	1	IP-10	τ(10)=-0.598, p=0.018
KC	0	IL-1b	τ(6)=-0.602, p=0.114
IL-10	1	IL-1a	τ(7)=-0.724, p=0.037
IL-2	0	IL-10	τ(10)=-0.728, p=0.025
TNF-a	1	IFNy	τ(7)=-0.739, p=0.061
RANTES	1	G-CSF	τ(6)=-0.867, p=0.015

TNF-a	0	IL-2	τ(10)=-1, p=0.003
IL-4	0	IL-2	τ(10)=-1, p=0.003
